# Coexistence of Redox‐Active Metal and Ligand Sites in Copper‐Based Two‐Dimensional Conjugated Metal–Organic Frameworks as Active Materials for Battery‐Supercapacitor Hybrid Systems

**DOI:** 10.1002/cssc.202401454

**Published:** 2024-12-19

**Authors:** Ahmad Bagheri, Sebastiano Bellani, Hossein Beydaghi, Zhiyong Wang, Ahiud Morag, Marilena I. Zappia, Jaya‐Kumar Panda, Samaneh Vaez, Valentina Mastronardi, Agnese Gamberini, Sanjay Balkrishna Thorat, Matteo Abruzzese, Lea Pasquale, Renhao Dong, Minghao Yu, Xinliang Feng, Francesco Bonaccorso

**Affiliations:** ^1^ Graphene Labs Istituto Italiano di Tecnologia via Morego 30 16163 Genoa Italy; ^2^ Center for Advancing Electronics Dresden (cfaed) & Faculty of Chemistry and Food Chemistry Technische Universität Dresden 01062 Dresden Germany; ^3^ BeDimensional S.p.A. Lungotorrente Secca 30R 16163 Genoa Italy; ^4^ Max Planck Institute of Microstructure Physics Weinberg 2 06120 Halle Germany; ^5^ Department of Applied Science and Technology (DISAT) Politecnico di Torino 10129 Torino Italy; ^6^ Materials Characterization Facility Istituto Italiano di Tecnologia Via Morego 30 16163 Genova Italy; ^7^ Current Affiliation: Department of Chemistry & Materials Innovation Institute for Life Sciences and Energy (MILES, HKU-SIRI) The University of Hong Kong Hong Kong 999077 China

**Keywords:** Batteries, Energy storage, Metal-organic frameworks (MOFs), Redox-active sites., Supercapacitors

## Abstract

Two‐dimensional (2D) conjugated metal‐organic frameworks (c‐MOFs) are promising materials for supercapacitor (SC) electrodes due to their high electrochemically accessible surface area coupled with superior electrical conductivity compared to traditional MOFs. In this work, porous and non‐porous HHB−Cu (HHB=hexahydroxybenzene), derived through surfactant‐assisted synthesis are studied as representative 2D c‐MOF models with different characteristics, showing diverse reversible redox reactions with Na^+^ and Li^+^ in aqueous (10 M NaNO_3_) and organic (1.0 M LiPF_6_ in ethylene carbonate and dimethyl carbonate) electrolytes, respectively. These redox activities were here deployed to design negative electrodes for hybrid SCs (HSCs), combining the battery‐like property of HHB−Cu at the negative electrode and the high capacitance and robust cyclic stability of activated carbon (AC) at the positive electrodes. In the organic electrolyte, porous HHB−Cu‐based HSC achieves a maximum cell specific capacity (C_s_) of 22.1 mAh g^−1^ at 0.1 A g^−1^, specific energy (Es) of 15.55 Wh kg^−1^ at specific power (Ps) of 70.49 W kg^−1^, and 77 % cyclic stability after 3000 gravimetric charge–discharge (GCD) cycles at 1 A g^−1^ (specific metrics calculated on the mass of both electrode materials). In the aqueous electrolyte, porous HHB−Cu‐based HSC displays a C_s_ of 13.9 mAh g^−1^ at 0.1 A g^−1^, Es of 6.13 Wh kg^−1^ at 44.05 W kg^−1^, and 72.3 % C_s_ retention after 3000 GCD cycles. The non‐porous sample, interesting for its superior electrical conductivity despite its limited surface area compared to its porous counterpart, shows lower Es performance but better rate capability compared to the porous one. This study indicates the potential of assembling a battery‐SC hybrid system by rationally exploiting the battery‐like behavior of 2D c‐MOFs and the electrochemical double‐layer capacitance of AC.

## Graphical abstract

Two‐dimensional conjugated metal‐organic frameworks (2D c‐MOFs), synthesized using a surfactant‐assisted method, are studied as negative electrodes for hybrid supercapacitors (HSCs). Both porous and non‐porous HHB−Cu (hexahydroxybenzene‐Cu) structures demonstrate reversible redox reactions with sodium and lithium ions in aqueous and organic electrolytes, emphasizing superior energy storage and cyclic stability, making them promising for battery‐supercapacitor hybrid systems.

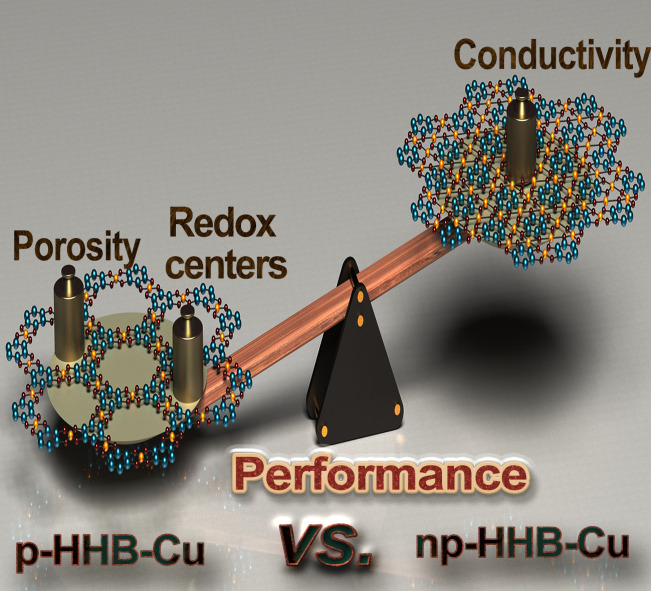

## 1 Introduction

Growing global demand for energy has motivated increasing efforts to develop advanced electrochemical energy storage devices to store electricity generated from renewable resources and to improve the power quality of grids by smoothing voltage fluctuations.[^1^, ^2^] Among a variety of energy storage systems, supercapacitors (SCs) offer excellent rate capability (*i. e*., fast charging and discharging rates), high maximum specific power (Ps>10 kW kg^−1^), wide range of operating temperatures (−40/+80 °C),^[3]^ and remarkable cyclic stability (up to millions of charge/discharge cycles).[^4^, ^5^] The SCs are classified into electrochemical double‐layer capacitors (EDLCs) and pseudocapacitors (PCs).[^6^, ^7^, ^8^, ^9^] The EDLCs rely on high specific surface area (SSA) materials (*i. e*., carbon‐based materials) as electrodes,[^10^, ^11^, ^12^] enabling charge storage through electrostatic processes, namely ion adsorption and desorption.[^7^, ^13^] Nevertheless, EDLCs face limitations in terms of energy density (Es), typically less than 10 Wh kg^‐1^ (at cell level).[^14^, ^15^, ^16^] In contrast, PCs store charge *via* rapid reversible electrochemical processes electrode‐electrolyte interface, like electrosorption, redox reactions, and intercalation, offering superior charge storage (*i. e*., superior specific capacity, C_s_) than EDLCs.[^1^, ^17^, ^18^] Utilizing pseudocapacitive electrodes in SCs is a practical method to reach Es of up to 50 Wh kg^−1^.[^19^, ^20^] Various inorganic materials, including TiO_2_, Nb_2_O_5_, MoS_2_, Fe_2_O_3,_ and MXenes have demonstrated appealing pseudocapacitive properties.[^6^, ^21^, ^22^] Nevertheless, their practical application is hindered by sluggish faradic reaction kinetics or structural instability,[^7^, ^19^, ^20^] as well as the use of bulky and heavy conductive substrates (*e. g*., foams) leading to unpractical configuration. In this context, hybrid SCs (HSCs) are devices combining a PC‐ or battery‐type electrode with an EDLC electrode enabling Faradic and non‐Faradic processes, respectively.[^6^, ^7^, ^8^, ^9^] Compared to EDLCs, HSC exhibit higher energy density and limited self‐discharge, while offering cycle life superior to those of metal‐ion batteries, sometimes solving dangerous thermal runaway issues.[^23^, ^24^] Thus, HSCs have the potential to bridge the gap between batteries and SCs in one physical unit.[^23^, ^24^] Recently, conjugated metal‐organic frameworks (c‐MOFs) have emerged as promising two‐dimensional (2D) materials for various applications, including electrochemical energy storage devices.[^14^, ^19^, ^25^] These materials possess pre‐designable structures, tunable pore sizes, and intrinsic electrical conductivity, superior to that of non‐conjugated MOFs.[^26^, ^27^, ^28^, ^29^] The electrical conductivity in c‐MOFs stems from the synergistic interaction between the 2D sheet components within the MOF structure, namely the ligands and metallic centers, which create conductive frameworks.[^30^, ^31^, ^32^]

The characteristics of c‐MOFs, including high porosity, large SSA, redox‐active sites, structural diversity, and versatile molecular design facilitated by both ligands and metallic centers, collectively enhance ion adsorption/desorption and reversible redox reactions, thereby improving their electrochemical performance.[^33^, ^34^]

Also, c‐MOFs possess highly order π‐stacks that provide open channels for fast ion diffusion, as needed for battery and SC electrode materials to achieve high‐rate capability devices.[^34^, ^35^] While porous c‐MOFs have been extensively studied to fabricate SC electrodes,[^19^, ^27^, ^36^, ^37^, ^38^] extensive research on the electrochemical properties of non‐porous c‐MOFs is still limited. The comparison of 2D c‐MOFs sharing the same chemistry but with different structural and electrical properties can provide valuable insights for their rational design of electrode materials for electrochemical energy storage applications.^[39]^ Notably, the 2D hexahydroxybenzene‐Cu (HHB−Cu) is a representative 2D c‐MOF that has been synthesized in porous and non‐porous structures (p‐HHB−Cu and np‐HHB−Cu, respectively).[^40^, ^41^, ^42^] The presence of redox‐active metal and ligand sites has been considered an interesting feature for electrocatalysis[^43^, ^44^, ^45^, ^46^] and electrochemical energy storage.[^40^, ^47^] In particular, p‐HHB−Cu has been proposed as an active material in Li‐ion battery electrodes.^[40]^ To overcome possible limitations, including insufficient electrical conductivity and excessive volume changes upon reversible redox reactions of 2D c‐MOFs compared to traditional electrode materials,^[48]^ carbonaceous materials (*e. g*., activated carbon (AC),[^49^, ^50^] graphene[^51^, ^52^, ^53^]) can be successfully incorporated into HSC electrodes to improve their rate capability and cyclic stability.[^49^, ^54^, ^55^] In addition, non‐porous 2D c‐MOFs may exhibit significantly different properties (*e. g*., electrical conductivity) compared to their porous counterparts, arising the importance of systematically extending studies on 2D c‐MOFs with different structural characteristics compared to porous ones. Based on these considerations, we characterized both p‐HHB−Cu and np‐HHB−Cu as the electrode materials for PCs in 10 M sodium nitrate (NaNO_3_) aqueous electrolyte and 1 m lithium hexafluorophosphate (LiPF_6_) in ethylene carbonate (EC) and dimethyl carbonate (DMC) (1 : 1 vol/vol), selected as representative aqueous and organic electrolytes, respectively. Cyclic voltammetry (CV), galvanostatic charge/discharge (GCD), and electrochemical impedance spectroscopy (EIS) characterizations were performed in a three‐electrode cell configuration on p‐HHB−Cu‐ and np‐HHB−Cu‐based electrodes to elucidate their electrochemical behavior in terms of charge transfer mechanisms, and determining their capacities. Afterward, HHB−Cu‐based electrodes were validated as negative electrodes in proof‐of‐concept HSCs using AC‐based positive electrodes. This study provides valuable insights into the electrochemical properties of 2D c‐MOFs, finding correlations with their structural characteristics, and their use in electrochemical energy storage devices.

## 2 Results and Discussion

**Figure 1:**
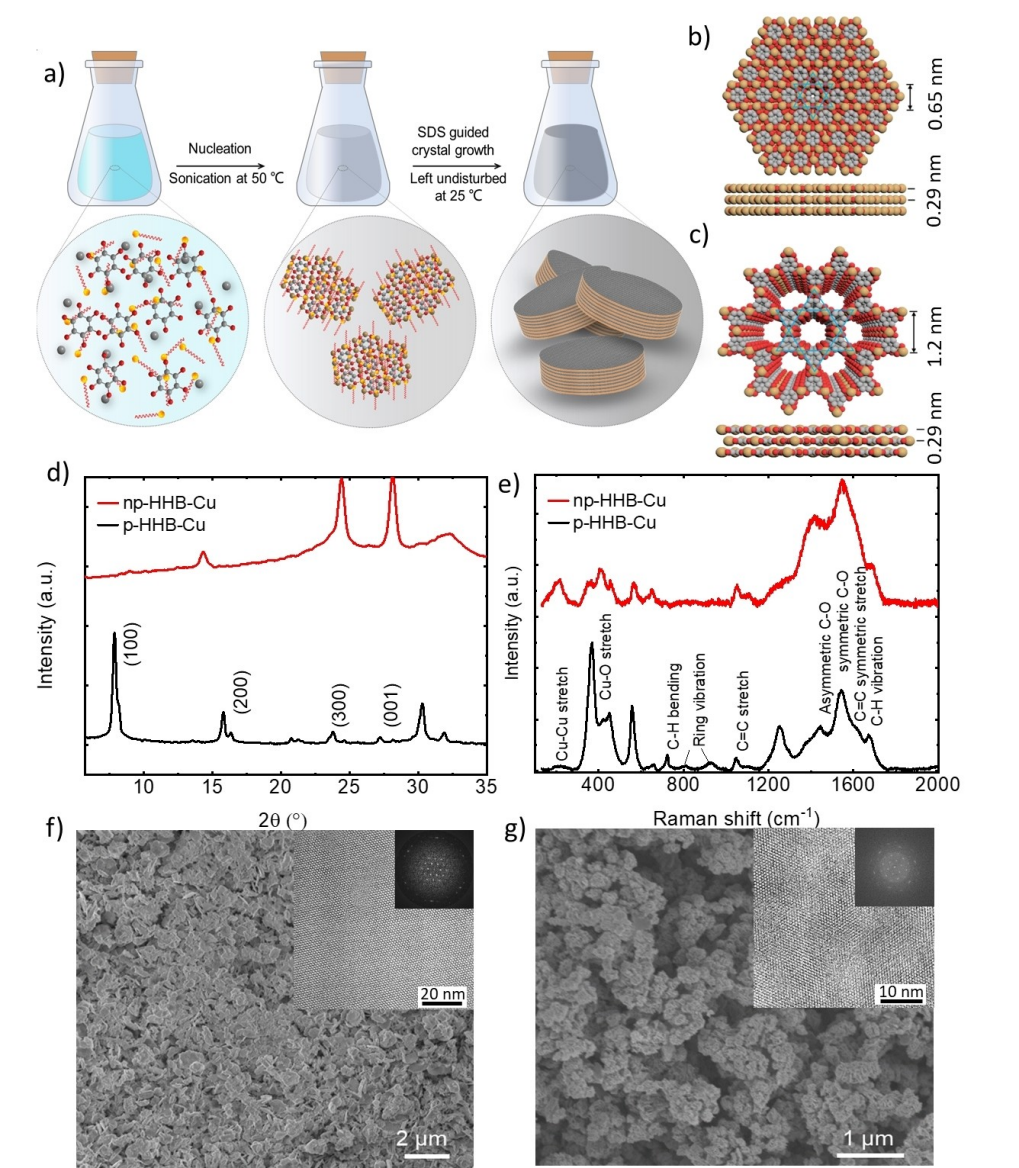
Synthesis procedure, sstructural and morphological characterization of p‐HHB−Cu and np‐HHB−Cu. a) Schematic illustration of the synthesis process for p‐HHB−Cu and np‐HHB−Cu. Unit cell structures of HHB−Cu derived using b) AA stacking model for np‐HHB−Cu and c) AB slipped‐parallel stacking model for p‐HHB−Cu. d) XRD patterns and e) Raman spectra measured for p‐HHB−Cu and np‐HHB−Cu. f,g) SEM images (insets are AC‐HRTEM images and the corresponding fast Fourier transform (FFT) images) of np‐HHB−Cu and p‐HHB−Cu, respectively.[^40^, ^41^]

Figure 1a depicts the surfactant‐assisted solution synthesis of p‐HHB−Cu and np‐HHB−Cu using an anionic surfactant, sodium dodecyl sulfate (SDS), as thoroughly discussed in previous studies.[^40^, ^41^] Figure 1b,c illustrate the structures of p‐HHB−Cu and np‐HHB−Cu. As shown in Figure 1d, the X‐ray diffraction (XRD) pattern measured for p‐HHB−Cu reveals distinct peaks at 2θ of 7.8°, 15.7° and 27.5°. These peaks correspond to the crystallographic planes (100), (200) and (300), respectively,[^26^, ^40^] confirming its crystalline structure with hexagonal in‐plane lattice (a=b=1.2 nm) and ordered stacking with an interlayer spacing of 2.88 Å. In contrast, the XRD pattern of np‐HHB−Cu displays (100), (110), (200) and (001) peaks at 2θ of 13.6°, 23.7°, 27.4° and 30.2°, respectively. These data indicate ordered hexagonal unit cells with a=b=0.75 nm and stacking with an interlayer spacing of ≈2.9 Å.^[26]^ Raman spectroscopy measurements (Figure 1e) were also carried out to assess the structural properties of p‐HHB−Cu and np‐HHB−Cu. The peaks at 205 cm^−1^ (p‐HHB−Cu) and 217 cm^−1^ (np‐HHB−Cu) correspond to Cu−Cu stretching modes, as assessed in previous studies.^[56]^ The peak at 450 cm^−1^ is related to the Cu−O stretching modes involving oxygen atoms linked with organic moieties in both np‐HHB−Cu and p‐HHB−Cu.^[57]^ Additional peaks include C−H bending at 725 cm^−1^, benzene ring breathing modes at 813 cm^−1^and 928 cm^−1^, and a C−H stretching mode at 1542 cm^−1^.[^58^, ^59^, ^60^] The peak at 1006 cm^−1^ is associated with the symmetric C=C stretching.^[57]^ The peaks at 1448 cm^−1^ and 1671 cm^−1^ indicate asymmetric and symmetric C−O stretching modes,^[59]^ respectively, associated with Cu−O bonds in the c‐MOF structures.^[60]^ Notably, while the C−C stretching, Cu−O and C−H bond intensities are similar in both np‐HHB−Cu and p‐HHB−Cu, differences in the Cu−O and Cu−Cu stretching bond intensities distinguish the porous and non‐porous structures.[^61^, ^62^, ^63^] Figure 1f,g show the scanning electron microscopy (SEM) images of p‐HHB−Cu and np‐HHB−Cu, in which the 2D crystal morphologies are recognizable. Aberration‐corrected high‐resolution transmission electron microscopy (AC‐HRTEM) images confirm a highly ordered hexagonal lattice, confirming the high crystallinity of p‐HHB−Cu nanosheets. The pore size of 1.2 nm measured from magnified AC‐HRTEM image aligns precisely with the structural model. The lateral dimensions range from 0.30 to 0.65 μm^2^. The electrical conductivity of p‐HHB−Cu and np‐HHB−Cu, determined through the van der Pauw method at 300 K and, was found to be 1.53×10^−7^ and 2.58×10^−2^ S cm^−1^, respectively (Table S1). The low conductivity measured for p‐HHB−Cu is consistent with a p‐type semiconducting behavior discussed in literature.[^40^, ^64^] Instead, the excellent electrical conductivity of np‐HHB−Cu is attributed to its enhanced density of Cu centers within the 2D plane. This increases charge carrier concentration and promotes overlap between Cu 3d orbitals and ligand π orbitals, providing electron transport pathways.[^40^, ^64^]

Based on N_2_ adsorption measurements at 77 K, p‐HHB−Cu demonstrates a Brunauer–Emmett–Teller (BET) SSA of 385 m^2^g^−1^. This high SSA is attributed to the exposure of the surface between between‐MOF layers, as also promoted by high crystallinity, large domain size, and nanoscale thickness.[^40^, ^41^]

**Figure 2:**
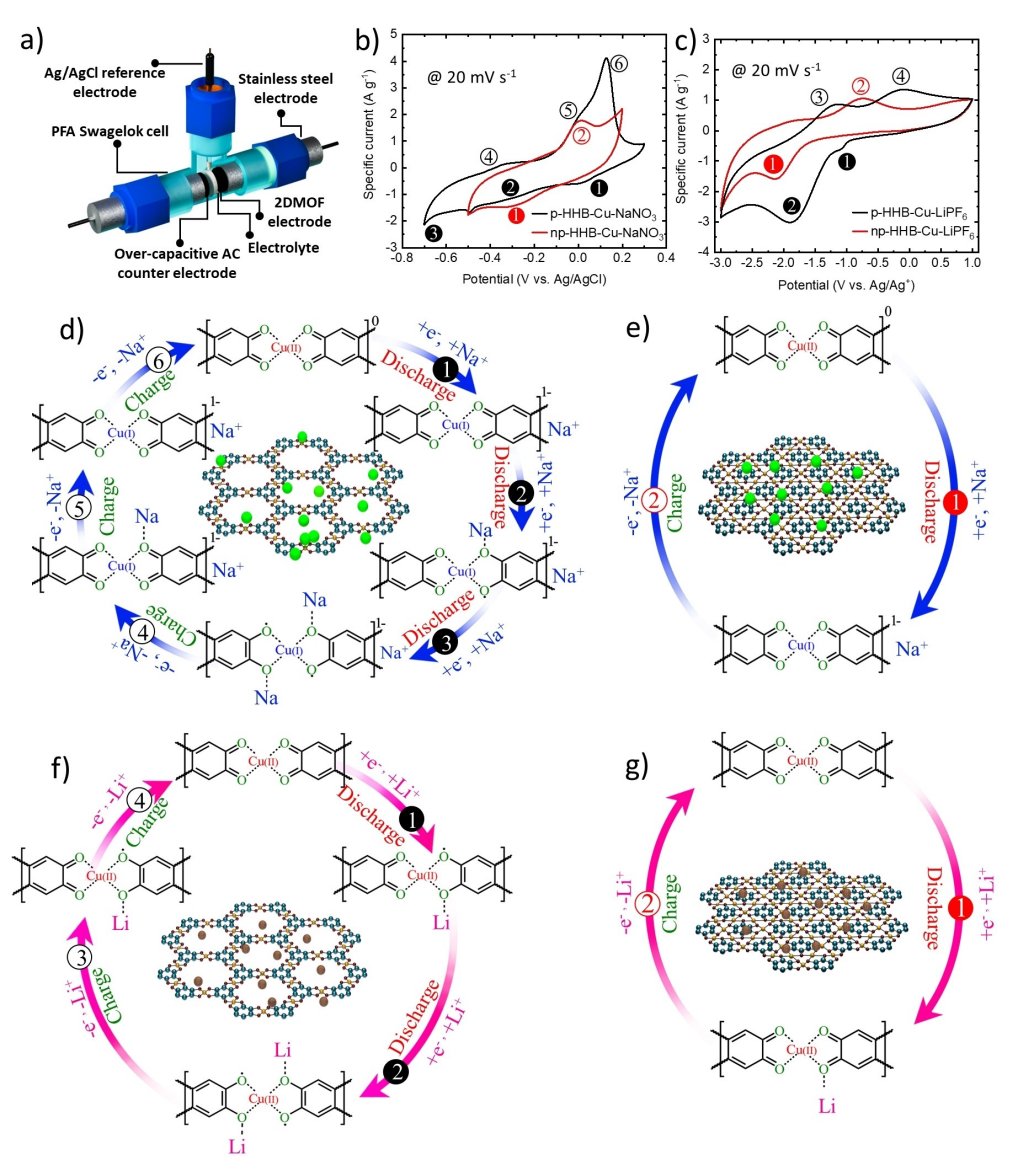
Electrochemical characterization of the investigated HHB−Cu‐based electrodes in different electrolytes. a) Schematic illustration of the three‐electrode Swagelok cell used for measurements. CV curves measured at a potential scan rate of 20 mV s^−1^ for b) p‐HHB−Cu‐NaNO_3_, and np‐HHB−Cu‐NaNO_3_, c) p‐HHB−Cu‐LiPF_6_, and np‐HHB−Cu‐LiPF_6_. Proposed charge storage mechanisms for d) p‐HHB−Cu‐NaNO_3_, e) np‐HHB−Cu‐NaNO_3_, f) p‐HHB−Cu‐LiPF_6_, and g) np‐HHB−Cu‐LiPF_6_. The mechanisms are depicted using a numbered sequence, highlighting key steps and the corresponding redox peaks observed in the cyclic voltammograms.

The electrochemical behavior of 2D c‐MOF‐based electrodes was assessed in a three‐electrode cell configuration, using p‐HHB−Cu or np‐HHB−Cu as the active material of the working electrode and either 10 m NaNO_3_ in water or 1 m LiPF_6_ in EC:DMC (1 : 1 vol/vol) as representative aqueous and organic electrolytes. Aqueous electrolytes typically offer higher ionic conductivity compared to organic ones, leading to high‐rate capability devices. This is advantageous for applications requiring rapid charging/discharging. However, aqueous electrolytes have a limited electrochemical stability window, typically up to 1.23 V (can be extended to 2 V), due to the occurrence of water splitting reactions.[^19^, ^50^, ^65^] This limits the voltage range and, consequently, the energy density of the corresponding SCs. Organic electrolytes have a broad electrochemical stability window, often exceeding 3 V. This enables higher operating voltages compared to those achieved with aqueous electrolyte, increasing the energy density.[^39^, ^66^] Figure 2a depicts the three‐electrode Swagelok cell configuration for electrochemical measurements of HHB−Cu‐based electrodes, with over‐capacitive AC as the counter electrode. The details of the electrode fabrication are reported in the Supporting Information (Experimental section). The investigated electrodes are named hereafter as X–Y, where X denotes the active material (p‐HHB−Cu or np‐HHB−Cu) while Y indicates the electrolyte salt (*i. e*., NaNO_3_ or LiPF_6_). The CV measurements at a potential scan rate of 20 mV s^−1^ first elucidated the redox behavior of p‐HHB−Cu‐ and np‐HHB−Cu‐based electrodes (Figure 2b,c). For both electrodes, the CV curves exhibit clear pairs of redox peaks, implying their battery‐like reversible faradaic behaviors usable in the cathode. Despite chemical similarities, p‐HHB−Cu and np‐HHB−Cu exhibit notable electrochemical differences. As shown in Figure 2b, compared to np‐HHB−Cu‐NaNO_3_, p‐HHB−Cu‐NaNO_3_ displays two additional pairs of redox peaks originated by the participation to Na^+^ (de)intercalation processes of unbonded electron pair of the oxygen atoms in the organic ligands.[^67^, ^68^] Specifically, the reduction peak at 0.00 V (*vs*. Ag/AgCl) refers to the Cu^2+^ reduction, while the subsequent peaks at −0.30 V and −0.5 V (*vs*. Ag/AgCl) correspond to the reduction of C=O bonds in the organic ligands.[^69^, ^70^, ^71^] The reduction process leads to the formation of C−O bonds, accompanied by the intercalation of Na^+^ ions.[^67^, ^68^, ^69^] The subsequent anodic peaks at −0.36 V and 0.01 V (*vs*. Ag/AgCl) signify the oxidation of the C−O bonds and the restoration of C=O bonds, leading to the extraction of Na^+^ ions. Additionally, the sharp peak observed at 0.13 V (*vs*. Ag/AgCl) is ascribed to the oxidation of Cu^+^.^[69]^ Overall, p‐HHB−Cu‐NaNO_3_ follows a three‐electron transfer mechanism (Figure 2d). Conversely, the oxygen atoms in np‐HHB−Cu are bonded to Cu^2+^, leading to an ion intercalation restricted between c‐MOF layers and leading to a single‐electron transfer (Figure 2e). Thus, the single oxidation peak at approximately 0.01 V (*vs*. Ag/AgCl) and a corresponding reduction peak at −0.35 V (*vs*. Ag/AgCl) are attributed to Cu^2+^/Cu^+^ ion redox reactions.[^70^, ^72^]

These results are consistent with previous literature reports.^[69]^ As shown in Figure 2c, the charge storage in p‐HHB−Cu‐LiPF_6_ is based on two‐electron transfer mechanism (Figure 2f), occurring over a broad operating potential windows (from −3.0 V to −1.0 V *vs*. Ag/Ag^+^). These results are in agreement with the literature,^[40]^ indicating the reversible transformation from neutral to negatively charged (reduced) states of HHB−Cu with Li^+^ intercalation.^[73]^ Differently from p‐HHB−Cu‐LiPF_6_, the charge storage process of np‐HHB−Cu‐LiPF_6_ follows a single‐electron mechanism (Figure 2g), indicating a Li^+^ (de)intercalation between c‐MOF layers, as for the case of Na^+^ intercalation. Despite their different charge storage mechanism, it should be noticed that the redox reactions at potentials higher than −0.50 V *vs*. Ag/Ag^+^ are hardly used in negative electrodes once combined with positive electrodes in full cells. In fact, 0.0 V *vs*. Ag/Ag^+^ is experimentally found between 3.50 V and 3.90 V *vs*. Li/Li^+^, depending on the electrolyte, thus negative electrodes are unlikely to operate at potentials higher than −0.50 V *vs*. Ag/Ag^+^.^[74]^ Therefore, hereafter, electrodes characterization in 1 m LiPF_6_ in EC:DMC (1 : 1 vol/vol) will be restricted to the practical potential window between −2.50 V and −0.50 V *vs*. Ag/Ag^+^.

Figure S1a–d show the CV curves measured for HHB−Cu‐based electrodes at a potential scan rate ranging from 0.5 to 50 mV s^−1^, revealing explicit redox peaks, and confirming further the battery‐like faradaic properties, especially at low potential scan rates. Importantly, the CV curves retain the redox peaks with increasing the potential scan rate, meaning fast charge transfer ability and suitable reversibility.^[75]^ By increasing the potential scan rate, the anodic and cathodic peaks shift towards higher and lower potentials, respectively, because of the polarization effect as well as the presence of electrodes internal resistance.[^48^, ^76^]

**Figure 3:**
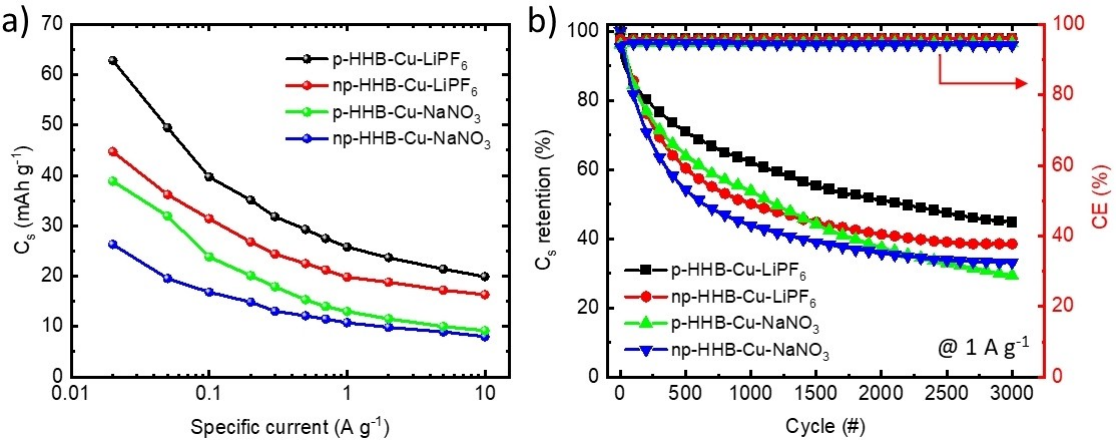
Electrochemical characterization of the HHB−Cu‐based electrodes. a) Electrode C_s_ vs. specific current plots acquired from GCD profile analysis at various specific currents, ranging from 0.02 to 10 A g^−1^. b) C_s_ retention and CE of electrodes over 3000 GCD cycles at 1 A g^−1^.

Figure S2a–d presents GCD profiles measured for p‐HHB−Cu‐NaNO_3_, np‐HHB−Cu‐NaNO_3_, p‐HHB−Cu‐LiPF_6,_ and np‐HHB−Cu‐LiPF_6_ electrodes, respectively, at specific currents ranging from 0.02 to 10 A g^−1^. All HHB−Cu‐based electrodes display non‐linear voltage‐time responses at various specific currents, confirming the battery‐like characteristics denoted by previous CV analyses. In both electrolytes examined, the HHB−Cu‐based electrodes show longer discharge times (and, therefore larger C_s_) than those measured for the np‐HHB−Cu‐based electrodes. Figure 3a shows C_s_ for all HHB−Cu‐based electrodes as a function of the specific current, as calculated from the GCD profiles (see Supporting Information, Equation (S1)). Notably, p‐HHB−Cu‐LiPF_6_ demonstrates the highest C_s_ (*e. g*., 62.8 mAh g^−1^ at 0.02 A g^−1^) amongst the investigated samples (at 0.02 A g^−1^, C_s_ of 44.7 mAh g^−1^ for np‐HHB−Cu‐LiPF_6_, 38.8 mAh g^−1^ for p‐HHB−Cu‐NaNO_3_ and 26.3 mAh g^−1^ for np‐HHB−Cu‐NaNO_3_). C_s_ decrease with increasing specific current, reaching 19.9, 16.3, 9.2, and 8.0 mAh g^−1^ at 10 A g^−1^ for p‐HHB−Cu‐LiPF_6_, np‐HHB−Cu‐LiPF_6_, p‐HHB−Cu‐NaNO_3_, and np‐HHB−Cu‐NaNO_3_, respectively.

This decrease of C_s_ with increasing the specific current is attributed to diffusion limitations of electrolyte ions within electrode pores and interlayer space.[^77^, ^78^] The np‐HHB−Cu‐LiPF_6_, better retain their C_s_, *i. e*., 36.5 %, with increasing the specific current from 0.02 to 10 A g^−1^ compared to the other samples (*i. e*., C_s_ retention of 31.7 %, 23.7 % and 30.4 % for p‐HHB−Cu‐LiPF_6_, p‐HHB−Cu‐NaNO_3_, and np‐HHB−Cu‐NaNO_3_, respectively). Despite the porous structures, p‐HHB−Cu may provide short ion transport pathways leading to rapid ion/electron diffusion.[^31^, ^79^, ^80^] Our data indicate that the superior electrical conductivity of np‐HHB−Cu compared to its porous counterpart (2.6×10^−2^ S cm^−1^ *vs*. 1.5×10^−7^ S cm^−1^) also plays a role in attaining elevated rate capability. The long‐term stability for HHB−Cu‐based electrodes was evaluated over 3000 GCD cycles at 1 A g^−1^. As shown in Figure 3b, p‐HHB−Cu‐LiPF_6_, np‐HHB−Cu‐LiPF_6_, p‐HHB−Cu‐NaNO_3_, and np‐HHB−Cu‐NaNO_3_ retained 44.7 %, 35.5 %, 29.4 %, and 33 % of their initial C_s_, respectively, with CE exceeding 90 % for all electrodes.

The electrochemical stability of representative p‐HHB−Cu‐NaNO_3_ and np‐HHB−Cu‐NaNO_3_ was investigated through XRD, Raman, and SEM measurements after 3000 GCD cycling. Figure S3 shows the XRD patterns of p‐HHB−Cu‐NaNO_3_, and np‐HHB−Cu‐NaNO_3_ before and after the cyclic stability test. The p‐HHB−Cu‐NaNO_3_ electrode exhibits main diffraction peaks at 23.8°, 35.4°, 42.5°, and 43.3°, while np‐HHB−Cu‐NaNO_3_ features peaks are at 22.8°, 36.5° and, 42.3°. These peaks correspond to the carbon cloth substrate (JCPDS No. 74–1162), Cu (JCPDS No. 98–005‐3247), and CuO (JCPDS No. 98–006‐9094), and Cu_2_O (98‐003‐8233). Importantly, the XRD patterns measured for the as‐produced samples exhibit sharp, well‐defined peaks, indicating highly crystalline and ordered structures. However, after the cyclic stability test, some changes were observed associated with the alteration of the structure of the MOF. This effect may result from repeated ion intercalation/deintercalation, causing mechanical stresses,^[39]^ *i. e*., expansion and contraction of the MOF during cycling.[^81^, ^82^, ^83^] Moreover, in the porous samples, some diffraction peaks, such as those at 13.4° and 29.4°, emerge after cycling, pointing out the formation of new crystalline phases.[^84^, ^85^] These new phases could result from side reactions, byproduct formation, or partial decomposition of the MOF into different compounds, such as copper oxides or hydroxides.[^86^, ^87^] By comparing the investigated samples, it is evident that the p‐HHB−Cu‐NaNO_3_ exhibits more pronounced changes in the XRD pattern after cycling. This is likely due to its larger surface area, which leads to more significant interactions with the electrolyte and, consequently, a greater extent of structural degradation.^[39]^

To further investigate the structural changes in HHB−Cu‐based electrodes after the GCD cycling, ex‐situ Raman spectroscopy was performed on both the p‐HHB−Cu‐NaNO_3_ and np‐HHB−Cu‐NaNO_3_ electrodes (Figure S4). Compared to pristine samples, Raman spectroscopy measurements of the samples after cycling reveal notable differences in both the metallic and organic regions, with several Raman bands exhibiting detectable peak shifts. These shifts, observed in the 1800–730 cm^−1^ range, predominantly correspond to modes associated with the organic framework of the MOF.[^88^, ^89^, ^90^] After 3000 GCD cycles, shifts in peak positions are evident for both samples, indicating changes in bond lengths or bond strengths, likely resulting from ion insertion/extraction during cycling or interactions between MOF and the electrolyte. These effects suggest the presence of mechanical stresses that strain the MOF structure, ultimately leading to a progressive capacity decay, as above discussed (Figure S4).[^90^, ^91^, ^92^] Specifically, significant changes are detected between 150 and 600 cm^−1^, where vibrational modes involving Cu^2+^ ions are prominent.[^88^, ^89^, ^90^] The peaks at 150–190 cm^−1^ and 239 cm^−1^ are attributed to stretching modes involving Cu–Cu dimers and Cu–O_w_ bonds, where O_w_ refers to the oxygen of water molecules adsorbed on the Cu^2+^, respectively.^[57]^ Additionally, the peaks in the 453–502 cm^−1^ range are related to Cu–O stretching modes involving the oxygen atoms of carboxylate bridges.[^63^, ^93^] The region from 1200 to 1700 cm^−1^, which corresponds to benzene‐ring‐related vibrations, shows clear evolution after cycling. In the HHB−Cu MOF structure, the potentials during charging may partially reduce the ligand within the MOF structure, altering the vibrational characteristics of the organic framework.[^19^, ^40^, ^94^] This reduction, together with repeated ion intercalation/deintercalation, likely induces structural degradation, contributing to capacity decay.[^36^, ^67^] Moreover, new peaks emerged in the Raman spectrum after cycling, indicating the formation of new chemical species. In particular, the characteristic peaks of monoclinic CuO appear around 296 cm^−1^ (A_g_ mode), 346 cm^−1^ (B_g_ mode), and 631 cm^−1^ (B_g_ mode).^[95]^ The formation of CuO is consistent with the XRD results. This oxidation process is a key factor in the structural degradation of MOFs, leading to the deterioration of the electrochemical performance over time.^[96]^ Compared to the np‐HHB−Cu‐NaNO_3_ samples, the p‐HHB−Cu‐NaNO_3_exhibits more pronounced changes in its Raman spectrum after cycling, including noticeable shifts in peak positions, broadening of peaks, and the emergence of new peaks. These changes are attributed to the large surface area of the porous MOF, which facilitates more extensive ion intercalation/deintercalation, leading to structural degradation and, thus, progressive capacity decay. In contrast, the np‐HHB−Cu‐NaNO_3_ shows less pronounced peak shifts and intensity changes. These observations are in accordance with the XRD results. Overall, the comparative analysis of the investigated electrodes indicates that the decay mechanism is more severe in the p‐HHB−Cu‐NaNO_3_. Figure S5 and Figure S6 report the post‐mortem SEM analyses of the p‐HHB−Cu‐NaNO_3_ and np‐HHB−Cu‐NaNO_3_ electrodes before and after GCD cycling, respectively. While XRD and Raman spectroscopy revealed significant structural changes in the HHB−Cu MOF samples after cyclic stability testing, SEM and mapping analyses did not show any noticeable morphological or compositional alterations. The SEM micrographs indicate that the material remains uniformly distributed, with no significant changes in particle morphology or aggregation after 3000 GCD cycles. Furthermore, the EDX maps of C, O, Cu, N, and Na, selected from specific areas of the electrodes, support that elements are evenly distributed across the material, with Na and N likely originating from residual NaNO_3_. Based on these results, we conclude that the electrochemical performance degradation of the electrode is more closely related to redox reaction‐driven structural changes of MOFs,[^67^, ^68^, ^69^, ^70^, ^97^] including crystallinity loss, bond reorganization, and the formation of new chemical species rather than external morphological alterations.

The impedance contributions from electrolytes and electrode‐electrolyte interfaces were assessed through EIS measurements. Specifically, the analysis of the complex impedance (Z) in a Nyquist plot (−Im[Z] *vs*. Re[Z]) provides insights into various electrochemical processes.^[98]^ Typically, the Nyquist plot of SC consists of three distinct frequency regions: high‐frequency redox, mid‐frequency diffusion, and low‐frequency capacitive reactions.^[99]^ Within the high‐frequency region, the plot reflects the electrical conductivity of the electrode as well as the redox charge transfer reactions occurring at the electrode‐electrolyte interface.[^100^, ^101^, ^102^] However, these contributions can overlap, making it challenging to extract accurate parameters. As shown in previous studies,[^98^, ^99^, ^103^, ^104^] the diameter of the semicircles in the Nyquist plot correlates with the interfacial resistance of the current collector/electrode interface.[^105^, ^106^, ^107^, ^108^] Meanwhile, the intersection of the Nyquist plot with the x‐axis (Z_re_‐axis) at the highest frequency provides the equivalent series resistance (R_s_), associated with the sum of the ionic resistance of the electrolyte and the electronic resistance of the electrodes.[^98^, ^103^, ^104^] Figure S7 illustrates the Nyquist plot of p‐HHB−Cu‐NaNO_3_, np‐HHB−Cu‐NaNO_3_, p‐HHB−Cu‐LiPF_6_ and np‐HHB−Cu‐LiPF_6_, recorded over a frequency range from 100 kHz to 10 mHz at open circuit potential with a voltage amplitude of 10 mV. The Nyquist plots are free of semicircles at high frequencies indicating negligible contact resistance at the current collector/electrode interface, while the low‐frequency region resembles a finite‐length Warburg element (Z_w_) associated with the non‐uniform pathway for ion transport from the bulk electrolyte to the porous electrode surface (resulting in a distributed charge storage), as well as to the so‐called “diffuse layer resistance”.^[109]^ The inset to Figure S7 shows that np‐HHB−Cu‐NaNO_3_ has the smallest R_s_ (0.26 Ω) amongst the investigated electrodes (0.42 Ω for p‐HHB−Cu‐NaNO_3_, 0.48 Ω for np‐HHB−Cu‐LiPF_6_, and 0.55 Ω for p‐HHB−Cu‐LiPF_6_). The p‐HHB−Cu‐LiPF_6_ displayed the largest |Im[Z]| amongst the electrodes, indicating a superior capacitive performance likely associated with the rapid electron ion transport in porous active materials.[^110^, ^111^]

The pseudocapacitive energy storage of the HHB−Cu active material relies on redox reactions involving charge transfer (Faradaic battery‐like behavior). This behavior overlaps with the surface‐limited process, leading to the EDLC capacitance. The predominance of one or another behavior can be determined by the analysis of CV curves at different potential scan rates.[^112^, ^113^] Figures S8–S11 (panels a) depict CV curves for HHB−Cu‐based electrodes recorded at different potential scan rates, from 1 to 10 mV s^−1^. The CV data at different potential scan rates were then analyzed using the power law Equation 1:

(1) $$i={av}^{b}$$

in which, i is the measured current (A), *v* is the scan rate (mV s^−1^), and a and *b* are the constant parameters, determined by the occurrence of capacitive and Faradaic processes. In particular, for diffusion‐controlled processes, the current response is proportional to the square root of the scan rate (*b*=0.5). Differently, the current response for a capacitive process is proportional to the scan rate (*b*=1).^[114]^ Figures S8–S11 (panels b) show the *b*‐values measured for the investigated electrodes as a function of their potential. Notably, *b*‐values of p‐HHB−Cu‐NaNO_3_ range from 0.55 to 0.84 (Figure S8b), confirming the occurrence of diffusion‐controlled processes in the potential range of −0.7 to 0.3 V *vs*. Ag/AgCl, which is consistent with previous CV and GCD analyses (Figures S1 and S2). Similarly, np‐HHB−Cu‐NaNO_3_ (Figure S9b), p‐HHB−Cu‐LiPF_6_ (Figure S10b), and np‐HHB−Cu‐LiPF_6_ (Figure S11b) exhibit *b*‐values ranging larger than 0.5, meaning the diffusion‐controlled processes are those determining the C_s_ of these active materials. To further analyze the capacitive contribution to the overall current response, the current response at a fixed potential was analyzed according to the following Equation 2:^[99]^

(2) $$i\;\left(V\right)=k_{1}\nu +k_{2}\nu^{1/2}$$

in which, *υ* is the scan rate (mV s^−1^) and *k*_1_ *υ* and *k*_2_ *υ* ^1/2^ represent the currents from surface capacitance contribution and the diffusion‐controlled faradaic processes, respectively.^[99]^ Equation (2) can also be rearranged as;

(3) $$i\;\left(V\right)/\nu^{1/2}=k_{1}\nu^{1/2}+k_{2}$$

Consequently, *k*_1_ and *k*_2_ can be derived from the linear plot of *i (V)*/*ν* ^1/2^ *vs. ν* ^1/2^. As reported in Figures S8–S11 (panels c) for p‐HHB−Cu‐NaNO_3_, np‐HHB−Cu‐NaNO_3_, p‐HHB−Cu‐LiPF_6_, and np‐HHB−Cu‐LiPF_6_, respectively, at a 5 mV s^−1^, diffusion‐controlled processes represent 53.9 %, 60.0 %, 46.2 %, and 56.4 % of total stored charge, respectively (panel d of Figures S8–S11). As expected, panels e and f of Figures S8–S11 demonstrate that the capacitive charge storage increases with increasing the potential scan rates, at which diffusion‐controlled faradaic processes are weakened due to insufficient kinetics of Faradaic reactions.[^36^, ^115^]

**Figure 4:**
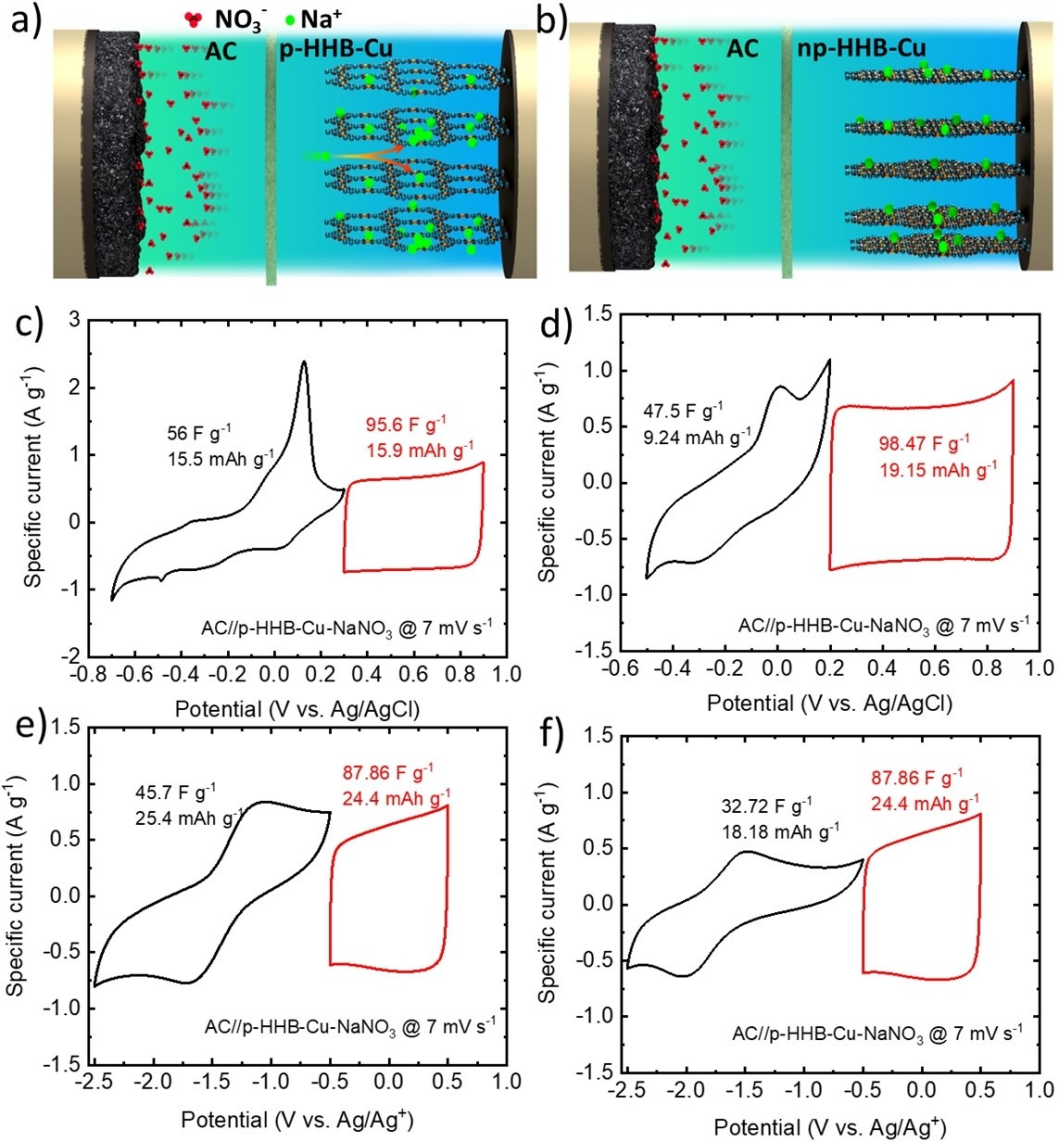
Electrochemical characterization of the investigated HHB−Cu‐based HSCs. a,b) Schematics of representative HSCs, i. e., AC//p‐HHB−Cu‐NaNO_3_, and AC//np‐HHB−Cu‐NaNO_3_, respectively_._ CV curves of AC and p‐HHB−Cu‐NaNO_3_, in c) AC//p‐HHB−Cu‐NaNO_3_, d) AC//np‐HHB−Cu‐NaNO_3_, e) AC//p‐HHB−Cu‐LiPF_6_, and f) AC//np‐HHB−Cu‐LiPF_6_, measured at 7 mV s^−1^ in a three‐electrode cell configuration.

To further validate the charge storage performances of HHB−Cu‐based electrodes in practical devices, HSCs were fabricated using 10 m NaNO_3_ in water and 1 M LiPF_6_ EC/DMC as electrolytes. Based on electrochemical characterization of HHB−Cu active materials in three‐electrode cell configurations, 2D c‐MOFs (p‐HHB−Cu or np‐HHB−Cu) were used as the negative electrode and AC was instead deployed as the positive electrode. The ratio between the masses of the electrode materials was adjusted to balance the C_s_ of negative and positive electrodes (see Supporting Information, Equation (S2)). Before fabricating HSCs, the electrochemical performance of the AC‐based electrode was assessed in a three‐electrode cell configuration (Figure S12). Figure 4a,b schematically show AC//p‐HHB−Cu‐NaNO_3_ and AC//np‐HHB−Cu‐NaNO_3_ HSC configurations. The working voltage windows (WVW) of the HSCs were determined by combining potential ranges from separate CV measurements at 7 mV s^−1^ (Figure 4c–f). In agreement with our previous electrochemical analyses, the CV curves of 2D c‐MOFs display redox peaks (Faradaic behavior), while the voltammograms of AC exhibit nearly rectangular shapes (capacitive behavior).

**Figure 5:**
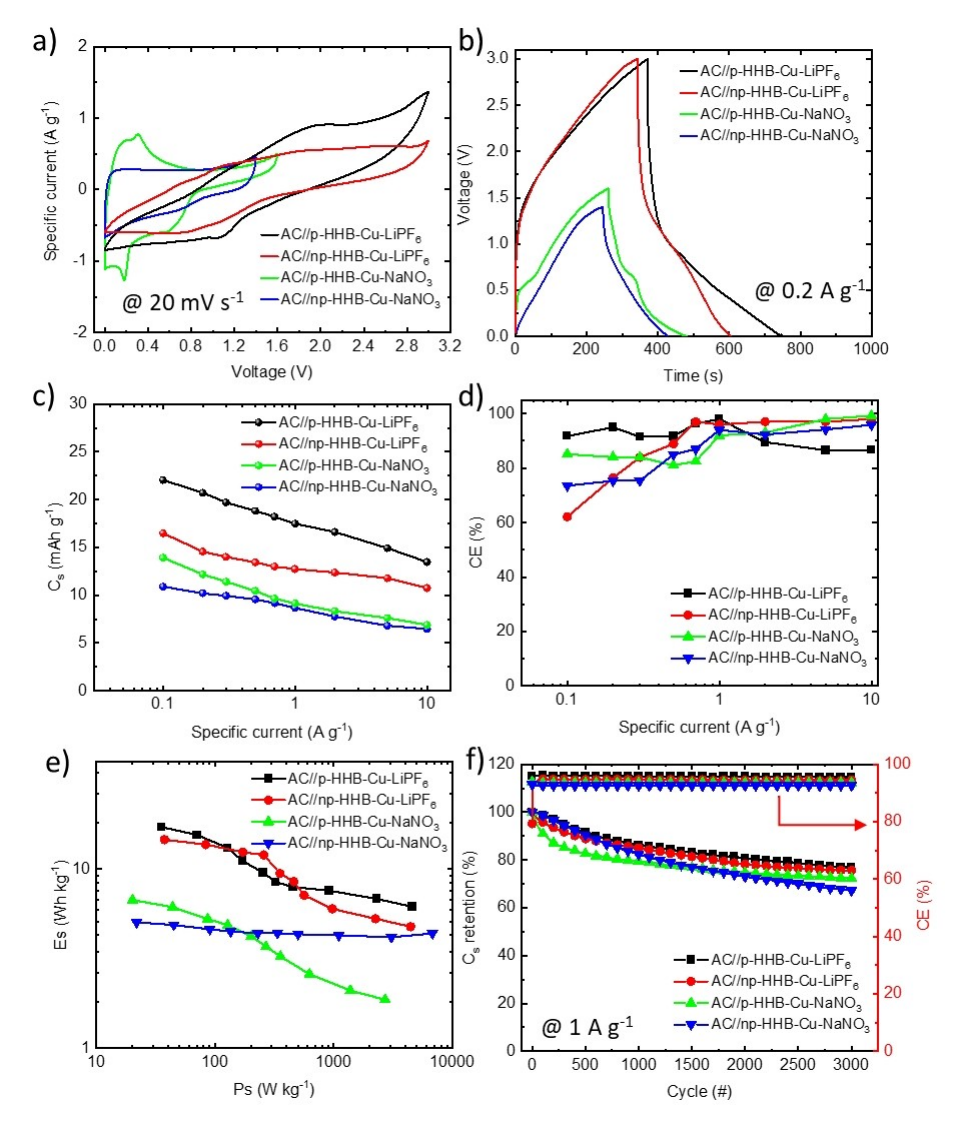
Electrochemical characterization of the investigated HHB−Cu‐based HSCs. a) CV curves, measured at 20 mV s^−1^, and b) GCD profiles, measured at 0.2 A g^−1^ for AC//p‐HHB−Cu‐NaNO_3_, AC//np‐HHB−Cu‐NaNO_3_, AC//p‐HHB−Cu‐LiPF_6_ and AC//np‐HHB−Cu‐LiPF_6_. c) C_s_ and d) CE as a function of the specific current (data extrapolated from the GCD profiles), e) Ragone plots and f) C_s_ retention and CE over 3000 GCD cycles at 1 A g^−1^, measured for the investigated HSCs.

Figure 5a shows the CV curves measured for the investigated HSCs, *i. e*., AC//p‐HHB−Cu‐NaNO_3,_ AC//np‐HHB−Cu‐NaNO_3_, AC//p‐HHB−Cu‐LiPF_6_, and AC//np‐HHB−Cu‐LiPF_6_ at a voltage scan rate of 20 mV s^−1^, using WVWs of 1.6, 1.4, and 3.0 V, respectively. The presence of redox peaks in the CV curves implies the co‐existence of capacitive and pseudocapacitive behaviors. As shown in Figures S13a–d, the oxidation and reduction peaks shift towards higher and lower potentials, respectively, because of the polarization effect as well as the internal resistance of the electrodes.[^116^, ^117^] Remarkably, even at 50 mV s^−1^, clear redox peaks indicate excellent kinetics of the pseudocapacitive (Faradaic) processes.[^54^, ^118^] For AC//p‐HHB−Cu‐NaNO_3_ and AC//np‐HHB−Cu‐NaNO_3_, the current response at low voltages (0–0.8 V) indicates a battery‐type behavior, whereas at higher voltages (*i. e*., >0.8 V), the behavior switches to an EDLC‐type one. Similarly, for the AC//p‐HHB−Cu‐LiPF_6_ and AC//np‐HHB−Cu‐LiPF_6_ devices, redox peaks are observed between 0.0 and 2.0 V, followed by a narrower current range at higher voltages (*i. e*., between 2.0–3.0 V), implying that both p‐HHB−Cu and np‐HHB−Cu exhibit a combination of capacitive and battery‐like behaviors even in the organic electrolyte. Similarly, for the AC//p‐HHB−Cu‐LiPF_6_ and AC//np‐HHB−Cu‐LiPF_6_ devices, redox peaks are observed between 0.0 and 2.0 V. This is followed by less intense current response at higher voltages (*i. e*., between 2.0–3.0 V). These results indicate that both p‐HHB−Cu and np‐HHB−Cu exhibit a combination of capacitive and battery‐like behaviors.

Figure 5b shows the GCD profiles for AC//p‐HHB−Cu‐NaNO_3_ and AC//np‐HHB−Cu‐NaNO_3_, AC//p‐HHB−Cu‐LiPF_6_ and np‐HHB−Cu‐LiPF_6_, measured at 0.2 A g^−1^. Figures S14a–d show the GCD profiles measured for the investigated electrodes at different specific currents. In general, the non‐linear shape of the GCD curves indicates the concomitance of Faradic and capacitive processes,[^1^, ^116^] as concluded by CV analysis. Figure 5c shows the C_s_ calculated from the GCD profiles, revealing that AC//p‐HHB−Cu‐LiPF_6_ achieved a maximum C_s_ of 22.1 mAh g^−1^ at 0.1 A g^−1^. Similarly, AC//np‐HHB−Cu‐LiPF_6_ exhibited a maximum C_s_ of 16.5 mAh g^−1^ at 0.1 A g^−1^, which is around 25 % lower than that of the AC//p‐HHB−Cu‐LiPF_6_. The superior performance of AC//p‐HHB−Cu‐LiPF_6_ compared to AC//p‐HHB−Cu‐LiPF_6_ is consistent with three‐electrode cell configuration data of the negative electrodes and is associated with the porous structure of p‐HHB−Cu.[^31^, ^66^] Similar trends were observed for the HSCs based on 10 m NaNO_3_ aqueous electrolyte. At 0.1 A g^−1^, the AC//p‐HHB−Cu‐NaNO_3_ and AC//np‐HHB−Cu‐NaNO_3_ HSCs exhibited C_s_ of 13.9 and 10.9 mAh g^−1^, respectively. For all the HSCs, C_s_ diminished with increasing the specific current, despite showing an interesting rate capability (*e. g*., at 10 A g^−1^, C_s_ retention was 61 % and 65.2 % for AC//p‐HHB−Cu‐LiPF_6_ and AC//np‐HHB−Cu‐LiPF_6_, respectively).^[47]^

At 10 A g^−1^, AC//p‐HHB−Cu‐LiPF_6_ HSC exhibited a C_s_ of 13.5 mAh g^−1^, corresponding to ≈61.1 % of the C_s_ measured at 0.1 A g^−1^, indicating a satisfactory rate capability.^[38]^ AC//np‐HHB−Cu‐LiPF_6_, AC//p‐HHB−Cu‐NaNO_3_ and AC//np‐HHB−Cu‐NaNO_3_ also exhibit notable C_s_ retention, highlighting a superior rate capability for devices based on np‐HHB−Cu likely due to their excellent electron conductivity.

Figures S15–S18 (panels a and b) show the CV curves and the corresponding *b*‐values measured for the investigated HSCs, screening voltage scan rate for 1 to 10 mV s^−1^. In agreement with three‐electrode cell configuration characterizations, *b*‐values between 0.5 and 1.0 indicate the co‐existence of Faradaic and capacitive processes.[^55^, ^119^] The extrapolation of k_1_ and k_2_ parameters from the analysis of CV curves (Equation (2) and (3)) (panels c of Figures S15–S18) allowed the contribution of capacitive and (Faradaic) diffusion‐controlled processes to the total C_s_ at different voltage scan rates, ranging from 1 to 10 mV s^−1^. At 5 mV s^−1^, Faradaic contributions accounted for 25.8 %, 33.7 %, 38.9 %, and 37.0 % for AC//p‐HHB−Cu‐NaNO_3_, AC//np‐HHB−Cu‐NaNO_3_, AC//p‐HHB−Cu‐LiPF_6_, and AC//np‐HHB−Cu‐LiPF_6_, respectively (panels d in Figures S15–S18). As shown in panels e and f in Figures S15–S18, the capacitive contribution gradually increases with increasing the voltage scan rate, as expected for HSCs.[^120^, ^121^] As shown in Figure 5d, the Coulombic efficiency (CE) measured for the HSCs surpasses 90 % at high specific currents (>1 A g^−1^). At lower specific currents (<1 A g^−1^), a decrease of CE likely occurs due to parasitic reactions, which are often observed in HSCs, especially those based on aqueous electrolytes.^[122]^ In Figure S19, the Nyquist plots of HSCs reveal that AC//np‐HHB−Cu‐NaNO_3_, AC//p‐HHB−Cu‐NaNO_3_, AC//np‐HHB−Cu‐LiPF_6_, and AC//p‐HHB−Cu‐LiPF_6_ displayed R_s_ of 0.23, 0.38, 1.78, and 1.92 Ω, respectively. In general, low R_s_ values are measured in aqueous electrolyte and for negative electrodes based on np‐HHB−Cu, which is consistent with previous electrochemical characterizations in three‐electrode cell configurations (Figure S7).^[123]^ At low frequencies (*i. e*., 0.01 to 100 Hz), AC//p‐HHB−Cu‐LiPF_6_ displayed the largest |Im[Z]| amongst the HSCs, confirming the superior capacitive performance p‐HHB−Cu‐LiPF_6_ among the investigate electrode/electrolyte systems.[^110^, ^124^] Figure 5e shows the Ragone plots (Es *vs*. Ps) measured for the investigated HSCs, as derived from the GCD profile analysis (see Supporting Information, Equations (S3) and (S4)). AC//p‐HHB−Cu‐LiPF_6_ achieved the highest Es of 15.55 Wh kg^−1^ at Ps of 70.5 W kg^−1^, followed by AC//np‐HHB−Cu‐LiPF_6_ with Es of 13.76 Wh kg^−1^ at 83.41 W kg^−1^. Lower values of Es are instead reached by AC//p‐HHB−Cu‐NaNO_3_ and AC//np‐HHB−Cu‐NaNO_3_, *i. e*., 6.13 Wh kg^−1^ at 44.05 W kg^−1^ and 4.88 Wh kg^−1^ at 44.93 W kg^−1^, respectively. The lower performance of HSCs based on aqueous electrolyte compared to those obtained with organic electrolyte are ascribed to the restricted WVW of aqueous electrolytes. The cyclic stability of HHB−Cu‐based HSCs was evaluated over 3000 GCD cycles at 1 A g^−1^. Figure 5f shows that AC//p‐HHB−Cu‐LiPF_6_ retained 77 % of its initial C_s_, while AC//np‐HHB−Cu‐LiPF_6_ exhibited 75.7 % C_s_ retention. After 3000 GCD cycles, CE was more than 95 % for both cases. For AC//p‐HHB−Cu‐NaNO_3_ and AC//np‐HHB−Cu‐NaNO_3_, C_s_ retention was 72.3 % and 67.2 %, with CE values of 93.6 % and 92.5 %, respectively.

## 3 Conclusions

This study highlights the potential of surfactant‐assisted solution‐derived HHB−Cu as active material of the negative electrode for hybrid supercapacitors (HSCs). Both p‐HHB−Cu‐ and np‐HHB−Cu were investigated to assess the role of their different porosity, specific surface area (SSA) and electrical conductivity on the electrochemical performance of the corresponding electrodes in both aqueous (10 m NaNO_3_ in water) and organic (1.0 m LiPF_6_ in EC/DMC) electrolytes. The charge storage mechanisms revealed a combination of capacitive (EDLC‐type) and Faradaic (battery‐type) behaviors, with pseudocapacitive effects retained even at high specific currents as 10 A g^−1^. In terms of C_s_, p‐HHB−Cu‐based electrodes outperformed those based on np‐HHB−Cu, achieving maximum C_s_ of 49.5 mAh g^−1^ at 0.1 A g^−1^ in 1.0 m LiPF_6_ in EC/DMC and 31.9 mAh g^−1^ in 10 m NaNO_3_, respectively. Afterward, p‐HHB−Cu‐ and np‐HHB−Cu‐based electrodes were validated as negative electrodes in HSCs using AC‐based positive electrodes. In particular, AC//p‐HHB−Cu‐NaNO_3_ achieved a C_s_ of 13.9 mAh g^−1^ at 0.1 A g^−1^, with Es of 6.13 Wh kg^−1^ at 44.05 W kg^−1^, together with promising cyclic stability (C_s_ retention of 72.3 % and final CE of 93.6 % after 3000 GCD cycles at 1 A g^−1^). AC//p‐HHB−Cu‐LiPF_6_ exhibited a specific capacity of 22.1 mAh g^−1^ at 0.1 A g^−1^, with Es of 15.55 Wh kg^−1^ at 70.5 W kg^−1^, while showing a C_s_ retention of 77 % with a final CE of 95.5 % after 3000 GCD cycles at 1 A g^−1^. During cycling, Cu^2+^ in CuO_4_ units within the MOF framework can be reduced to Cu^+^ or Cu^0^, then re‐oxidize to Cu^2+^. This redox cycling distorts and breaks down CuO_4_ units, weakening the crystal structure. Raman shifts indicate changes in bond lengths and strengths, reflecting altered Cu ion coordination. Organic ligands, particularly catecholates from hexahydroxybenzene, oxidize to benzoquinones and may revert, but irreversible changes weaken the coordination network, leading to capacity decay. The p‐HHB−Cu materials degrade more severely due to their larger surface area, which increases parasitic reactions with electrolytes. Overall, our results elucidate the electrochemical behaviors of representative porous and non‐porous 2D c‐MOFs, sharing the same chemistry but different structural, morphological, and electrical properties. Our electrochemical data mainly indicate that porous structures with high SSA are favorable to obtaining high C_s_ (and, thus, high Es), while the superior conductivity of non‐porous structures may be the key to minimizing R_s_, which is pivotal to reaching high‐rate capability properties. These findings provide valuable insights into the role of 2D c‐MOFs for advanced energy storage applications, guiding the effective exploration of multielectron Faradaic reactions for advanced HSCs.

## 4 Experimental Section

Details of the methods are presented in Supporting Information.

## Conflict of Interests

A.B., S.B., H.B., M.I.Z., J.K. P., S.V., V. M., S.B.T., and M.A are employees of BeDimensional S.p.A., a company producing and commercializing 2D crystals, while F.B. is its co‐founder, Scientific Director and board member. The other authors declare no conflict of interest.

5

## Supporting information

As a service to our authors and readers, this journal provides supporting information supplied by the authors. Such materials are peer reviewed and may be re‐organized for online delivery, but are not copy‐edited or typeset. Technical support issues arising from supporting information (other than missing files) should be addressed to the authors.

Supporting Information

## Data Availability

The data that support the findings of this study are available from the corresponding author upon reasonable request.

## References

[1] L. Qiu , W. Yang , Q. Zhao , S. Lu , X. Wang , M. Zhou , B. Tao , Q. Xie , Y. Ruan , ACS Appl. Nano Mater. 2022, 5, 6192–6200.

[2] A. Bagheri , P. Salarizadeh , M. S. A. Hazer , P. Hosseinabadi , S. Kashefi , H. Beydaghi , Electrochim. Acta 2019, 295, 875–890.

[3] M. A. Garakani , S. Bellani , V. Pellegrini , R. Oropesa-Nuñez , A. E. D. R. Castillo , S. Abouali , L. Najafi , B. Martín-García , A. Ansaldo , P. Bondavalli , C. Demirci , V. Romano , E. Mantero , L. Marasco , M. Prato , G. Bracciale , F. Bonaccorso , Energy Storage Mater. 2021, 34, 1–11.

[4] A. Chaichi , G. Venugopalan , R. Devireddy , C. Arges , M. R. Gartia , ACS Appl. Energy Mater. 2020, 3, 5693–5704.

[5] G. Jiang , J. Cai , M. Krishnamoorthy , R. A. Senthil , Y. Sun , X. Li , J. Pan , ACS Appl. Energy Mater. 2022, 5, 4138–4148.

[6] M. Najafi , S. Bellani , V. Galli , M. I. Zappia , A. Bagheri , M. Safarpour , H. Beydaghi , M. Eredia , L. Pasquale , R. Carzino , S. Lauciello , J. K. Panda , R. Brescia , L. Gabatel , V. Pellegrini , F. Bonaccorso , Electrochem 2022, 3, 463–478.

[7] A. Afif , S. M. Rahman , A. Tasfiah Azad , J. Zaini , M. A. Islam , A. K. Azad , J. Storage Mater. 2019, 25, 100852.

[8] M. Z. Iqbal , U. Aziz , J. Storage Mater. 2022, 46, 103823.

[9] N. R. Chodankar , H. D. Pham , A. K. Nanjundan , J. F. S. Fernando , K. Jayaramulu , D. Golberg , Y. K. Han , D. P. Dubal , Small 2020, 16, 1–35. 10.1002/smll.202002806 32761793

[10] A. Bagheri , S. Bellani , H. Beydaghi , M. Eredia , L. Najafi , G. Bianca , M. I. Zappia , M. Safarpour , M. Najafi , E. Mantero , Z. Sofer , G. Hou , V. Pellegrini , X. Feng , F. Bonaccorso , ACS Nano 2022, 16, 16426–16442. 36194759 10.1021/acsnano.2c05640 PMC9620411

[11] Y. Zhu , S. Murali , M. D. Stoller , K. J. Ganesh , W. Cai , P. J. Ferreira , A. Pirkle , R. M. Wallace , K. A. Cychosz , M. Thommes , D. Su , E. A. Stach , R. S. Ruoff , Science 2011, 332, 1537–1541. 21566159 10.1126/science.1200770

[12] F. Bonaccorso , L. Colombo , G. Yu , M. Stoller , V. Tozzini , A. C. Ferrari , R. S. Ruoff , V. Pellegrini , Science 2015, 347, 1246501. 25554791 10.1126/science.1246501

[13] L. M. Da Silva , R. Cesar , C. M. R. Moreira , J. H. M. Santos , L. G. De Souza , B. M. Pires , R. Vicentini , W. Nunes , H. Zanin , Energy Storage Mater. 2020, 27, 555–590.

[14] Z. Gan , J. Yin , X. Xu , Y. Cheng , T. Yu , ACS Nano 2022, 16, 5131–5152. 35293209 10.1021/acsnano.2c00557

[15] X. Yang , T. Lv , J. Qiu , Small 2023, 19, 1–32. 10.1002/smll.202300336 36840663

[16] E. Pomerantseva, F. Bonaccorso, X. Feng, Y. Cui, Y. Gogotsi, *Science* **2019**, *366*, eaan8285. 10.1126/science.aan8285 31753970

[17] C. Cong , H. Ma , Small 2023, 19, 1–37. 10.1002/smll.202207547 36631286

[18] M. Gentile , S. Bellani , M. I. Zappia , A. Gamberini , V. Mastronardi , M. Abruzzese , L. Gabatel , L. Pasquale , S. Marras , A. Bagheri , H. Beydaghi , E. L. Papadopoulou , G. Lanzani , F. Bonaccorso , ACS Appl. Mater. Interfaces 2024, 16, 13706–13718. 38458613 10.1021/acsami.3c18629 PMC10958450

[19] M. R. Lukatskaya , D. Feng , S. M. Bak , J. W. F. To , X. Q. Yang , Y. Cui , J. I. Feldblyum , Z. Bao , ACS Nano 2020, 14, 15919–15925. 33166110 10.1021/acsnano.0c07292

[20] G. Wu , X. Wu , X. L. Zhu , J. Xu , N. Bao , ACS Nano 2022, 16, 10130–10155. 35839097 10.1021/acsnano.2c02841

[21] M. Hu , H. Zhang , T. Hu , B. Fan , X. Wang , Z. Li , Chem. Soc. Rev. 2020, 49, 6666–6693. 32781463 10.1039/d0cs00175a

[22] J. Ding , W. Hu , E. Paek , D. Mitlin , Chem. Rev. 2018, 118, 6457–6498. 29953230 10.1021/acs.chemrev.8b00116

[23] D. Ren , X. Li , X. Zhao , B. Liu , Z. Yang , J. He , T. Li , P. Pan , Appl. Energy 2022, 324, 119730.

[24] R. K. Devi , M. Ganesan , T. W. Chen , S. M. Chen , M. Akilarasan , S. P. Rwei , J. Yu , T. Elayappan , A. Shaju , J. Alloys Compd. 2023, 944, 169261.

[25] J. Huang , K. Yuan , Y. Chen , Adv. Funct. Mater. 2022, 32, 1–68. 10.1002/adfm.202204692 PMC9349794 35942272

[26] J. Park , A. C. Hinckley , Z. Huang , D. Feng , A. A. Yakovenko , M. Lee , S. Chen , X. Zou , Z. Bao , J. Am. Chem. Soc. 2018, 140, 14533–14537. 30176142 10.1021/jacs.8b06666

[27] M. Z. Iqbal , M. Shaheen , M. W. Khan , S. Siddique , S. Farid , S. Aftab , S. M. Wabaidur , Mater. Today Sustainability 2023, 22, 100331.

[28] P. Zhang , M. Wang , Y. Liu , Y. Fu , M. Gao , G. Wang , F. Wang , Z. Wang , G. Chen , S. Yang , Y. Liu , R. Dong , M. Yu , X. Lu , X. Feng , J. Am. Chem. Soc. 2023, 145, 6247–6256. 36893495 10.1021/jacs.2c12684

[29] A. Schneemann , R. Dong , F. Schwotzer , H. Zhong , I. Senkovska , X. Feng , S. Kaskel , Chem. Sci. 2021, 12, 1600–1619. 10.1039/d0sc05889k PMC8179301 34163921

[30] J. Lu , H. Duan , Y. Zhang , G. Zhang , Z. Chen , Y. Song , R. Zhu , H. Pang , ACS Appl. Mater. Interfaces 2022, 14, 25878–25885. 35618261 10.1021/acsami.2c02268

[31] J. Liu , Y. Zhou , Z. Xie , Y. Li , Y. Liu , J. Sun , Y. Ma , O. Terasaki , L. Chen , Angew. Chem. Int. Ed. 2020, 59, 1081–1086. 10.1002/anie.201912642 31674098

[32] H. Banda , J. Dou , T. Chen , Y. Zhang , M. Dincă , Angew. Chem. 2021, 133, 27325–27331. 10.1002/anie.202112811 34597446

[33] M. Majumder , M. S. Santosh , R. Viswanatha , A. K. Thakur , D. P. Dubal , K. Jayaramulu , Energy Storage Mater. 2021, 37, 396–416.

[34] L. Feng , K. Y. Wang , G. S. Day , M. R. Ryder , H. C. Zhou , Chem. Rev. 2020, 120, 13087–13133. 33049142 10.1021/acs.chemrev.0c00722

[35] G. Chakraborty , I. H. Park , R. Medishetty , J. J. Vittal , Chem. Rev. 2021, 121, 3751–3891. 33630582 10.1021/acs.chemrev.0c01049

[36] P. Zhang , M. Wang , Y. Liu , S. Yang , F. Wang , Y. Li , G. Chen , Z. Li , G. Wang , M. Zhu , R. Dong , M. Yu , O. G. Schmidt , X. Feng , J. Am. Chem. Soc. 2021, 143, 10168–10176. 34185519 10.1021/jacs.1c03039

[37] K. Chen , S. Zhao , J. Sun , J. Zhou , Y. Wang , K. Tao , X. Xiao , L. Han , ACS Appl. Energy Mater. 2021, 4, 9534–9541.

[38] Y. Wang , Y. Liu , H. Wang , W. Liu , Y. Li , J. Zhang , H. Hou , J. Yang , ACS Appl. Energy Mater. 2019, 2, 2063–2071.

[39] H. Banda , J. H. Dou , T. Chen , N. J. Libretto , M. Chaudhary , G. M. Bernard , J. T. Miller , V. K. Michaelis , M. Dincǎ , J. Am. Chem. Soc. 2021, 143, 2285–2292. 33525869 10.1021/jacs.0c10849

[40] Z. Wang , G. Wang , H. Qi , M. Wang , M. Wang , S. W. Park , H. Wang , M. Yu , U. Kaiser , A. Fery , S. Zhou , R. Dong , X. Feng , Chem. Sci. 2020, 11, 7665–7671. 34094144 10.1039/d0sc01408g PMC8159486

[41] M. Gao , Z. Wang , Z. Liu , Y. Huang , F. Wang , M. Wang , S. Yang , J. Li , J. Liu , H. Qi , P. Zhang , X. Lu , X. Feng , Adv. Mater. 2023, 35, 2305575. 10.1002/adma.202305575 37608530

[42] Z. Wang , P. St. Petkov , J. Zhang , B. Liang , S. Revuelta , K. Xiao , K. Tiwari , Q. Guo , Z. Li , J. Zhang , H. Qi , S. Zhou , U. Kaiser , T. Heine , E. Cánovas , S. S. P. Parkin , X. Feng , R. Dong , Adv. Funct. Mater. 2024, 2404680.

[43] L. Kong , M. Zhong , W. Shuang , Y. Xu , X. H. Bu , Chem. Soc. Rev. 2020, 49, 2378–2407. 32154522 10.1039/c9cs00880b

[44] L. M. Cao , J. Zhang , L. W. Ding , Z. Y. Du , C. T. He , J. Energy Chem. 2022, 68, 494–520.

[45] Q. Wang , Z. Zhang , X. Zhao , J. Xiao , D. Manoj , F. Wei , F. Xiao , H. Wang , S. Wang , ChemElectroChem 2020, 7, 289–298.

[46] H. Zhang , J. Su , K. Zhao , L. Chen , ChemElectroChem 2020, 7, 1805–1824.

[47] D. Sheberla , J. C. Bachman , J. S. Elias , C. J. Sun , Y. Shao-Horn , M. Dincǎ , Nat. Mater. 2017, 16, 220–224. 27723738 10.1038/nmat4766

[48] X. Shen , X. Wei , T. Wang , S. Li , H. Li , Chem. Eng. J. 2023, 461, 141745.

[49] M. Zhang , W. Zhou , X. Yan , X. Huang , S. Wu , J. Pan , Z. Shahnavaz , T. Li , X. Yu , Fuel 2023, 333, 126323.

[50] A. Bagheri , S. Taghavi , S. Bellani , P. Salimi , H. Beydaghi , J. K. Panda , M. Isabella Zappia , V. Mastronardi , A. Gamberini , S. Balkrishna Thorat , M. Abruzzese , L. Pasquale , M. Prato , M. Signoretto , X. Feng , F. Bonaccorso , Chem. Eng. J. 2024, 496, 153529.

[51] Y. Ma , H. Chang , M. Zhang , Y. Chen , Adv. Mater. 2015, 27, 5296–5308. 26293692 10.1002/adma.201501622

[52] T. Kshetri , D. T. Tran , D. C. Nguyen , N. H. Kim , K. tak Lau , J. H. Lee , Chem. Eng. J. 2020, 380, 122543.

[53] D. P. Chatterjee , A. K. Nandi , J. Mater. Chem. A 2021, 9, 15880–15918.

[54] S. E. Berrabah , A. Benchettara , F. Smaili , A. Benchettara , A. Mahieddine , J. Alloys Compd. 2023, 942, 169112.

[55] Y. Qiu , Z. Liu , Y. Sun , C. Wang , C. J. Barrow , J. M. Razal , W. Yang , L. Cui , J. Liu , ACS Appl. Mater. Interfaces 2022, 14, 34770–34780. 35867520 10.1021/acsami.2c08546

[56] B. Wang , J. Jin , B. Ding , X. Han , A. Han , J. Liu , Front. Mater. 2020, 7, 1–7.

[57] M. Todaro , A. Alessi , L. Sciortino , S. Agnello , M. Cannas , F. M. Gelardi , G. Buscarino , J. Spectrosc. 2016, 2016, 8074297.

[58] S. Leubner , V. E. G. Bengtsson , K. Synnatschke , J. Gosch , A. Koch , H. Reinsch , H. Xu , C. Backes , X. Zou , N. Stock , J. Am. Chem. Soc. 2020, 142, 15995–16000. 32820922 10.1021/jacs.0c06978

[59] Z. Zheng , L. Opilik , F. Schiffmann , W. Liu , G. Bergamini , P. Ceroni , L. T. Lee , A. Schütz , J. Sakamoto , R. Zenobi , J. Vandevondele , A. D. Schlüter , J. Am. Chem. Soc. 2014, 136, 6103–6110. 24673195 10.1021/ja501849y

[60] R. Nivetha , A. Sajeev , A. Mary Paul , K. Gothandapani , S. Gnanasekar , G. Jacob , R. Sellappan , V. Raghavan , N. K. Chandar , S. Pitchaimuthu , S. K. Jeong , A. Nirmala Grace , Mater. Res. Express 2020, 7, 114001.

[61] X. Cong , X. L. Liu , M. L. Lin , P. H. Tan , NPJ 2D Mater. Appl. 2020, 4, 1–12.

[62] A. E. J. Hoffman , L. Vanduyfhuys , I. Nevjestić , J. Wieme , S. M. J. Rogge , H. Depauw , P. Van Der Voort , H. Vrielinck , V. Van Speybroeck , J. Phys. Chem. C 2018, 122, 2734–2746. 10.1021/acs.jpcc.7b11031 PMC5808359 29449906

[63] K. Metavarayuth , O. Ejegbavwo , G. McCarver , M. L. Myrick , T. M. Makris , K. D. Vogiatzis , S. D. Senanayake , O. M. Manley , A. M. Ebrahim , A. I. Frenkel , S. Hwang , T. Rajeshkumar , J. D. Jimenez , K. Chen , N. B. Shustova , D. A. Chen , J. Phys. Chem. Lett. 2020, 11, 8138–8144. 32894952 10.1021/acs.jpclett.0c02539

[64] M. Wang , R. Dong , X. Feng , Chem. Soc. Rev. 2021, 50, 2764–2793. 33465213 10.1039/d0cs01160f

[65] L. Wang , K. W. Huang , J. Chen , J. Zheng , Sci. Adv. 2019, 5, 1–10. 10.1126/sciadv.aax4279 PMC6984968 32047853

[66] Q. Jiang , P. Xiong , J. Liu , Z. Xie , Q. Wang , X. Q. Yang , E. Hu , Y. Cao , J. Sun , Y. Xu , L. Chen , Angew. Chem. Int. Ed. 2020, 59, 5273–5277. 10.1002/anie.201914395 31893570

[67] Y. Chen , M. Tang , Y. Wu , X. Su , X. Li , S. Xu , S. Zhuo , J. Ma , D. Yuan , C. Wang , W. Hu , Angew. Chem. 2019, 131, 14873–14881. 10.1002/anie.201908274 31381218

[68] J. Park , M. Lee , D. Feng , Z. Huang , A. C. Hinckley , A. Yakovenko , X. Zou , Y. Cui , Z. Bao , J. Am. Chem. Soc. 2018, 140, 10315–10323. 30041519 10.1021/jacs.8b06020

[69] Y. Chen , Q. Zhu , K. Fan , Y. Gu , M. Sun , Z. Li , C. Zhang , Y. Wu , Q. Wang , S. Xu , J. Ma , C. Wang , W. Hu , Angew. Chem. Int. Ed. 2021, 60, 18769–18776. 10.1002/anie.202106055 34137139

[70] L. Wang , Y. Ni , X. Hou , L. Chen , F. Li , J. Chen , Angew. Chem. Int. Ed. 2020, 59, 22126–22131. 10.1002/anie.202008726 32812334

[71] Z. Wang , Y. Zhang , H. Jiang , C. Wei , Y. An , L. Tan , S. Xiong , J. Feng , Nano Res. 2023, 16, 458–465.

[72] G. Zhu , L. Chen , T. Lu , L. Zhang , M. S. A. Hossain , M. A. Amin , Y. Yamauchi , Y. Li , X. Xu , L. Pan , Environ. Res. 2022, 210, 112909. 35157915 10.1016/j.envres.2022.112909

[73] S. Tang , H. Ge , Z. Hao , X. He , H. Li , D. Trefilov , Y. Song , Y. Li , X. Fu , Y. Cui , Y. Chen , ACS Nano 2020, 14, 12719–12731. 32936616 10.1021/acsnano.0c02973

[74] K. Izutsu , in Electrochemistry in Nonaqueous Solutions: Second Edition, John Wiley & Sons, Hoboken, NJ 2009.

[75] X. Zhang , Q. Yin , F. Cao , Y. Wang , N. Liu , J. Liu , R. Liu , Appl. Surf. Sci. 2023, 616, 156533.

[76] X. Guan , J. Chen , E. Zhu , P. Yin , L. Yang , X. Guan , G. Wang , J. Mater. Sci. Technol. 2023, 150, 145–158.

[77] C. L. Wu , D. H. Chen , J. Alloys Compd. 2021, 872, 159702.

[78] Z. X. Li , B. L. Yang , K. Y. Zou , L. Kong , M. L. Yue , H. H. Duan , Carbon 2019, 144, 540–548.

[79] X. Zhang , J. Wang , X. Ji , Y. Sui , F. Wei , J. Qi , Q. Meng , Y. Ren , Y. He , D. Zhuang , J. Mater. Sci. Mater. Electron. 2020, 31, 16260–16268.

[80] A. M. Mohamed , A. O. Abo El Naga , T. Zaki , H. B. Hassan , N. K. Allam , ACS Appl. Energy Mater. 2020, 3, 8064–8074.

[81] X. Ou , X. Liang , F. Zheng , P. Wu , Q. Pan , X. Xiong , C. Yang , M. Liu , Electrochim. Acta 2017, 258, 1387–1396.

[82] Y. C. Lu , A. N. Mansour , N. Yabuuchi , Y. Shao-Horn , Chem. Mater. 2009, 21, 4408–4424.

[83] Y. Huang , F. M. Jin , F. J. Chen , L. Chen , J. Power Sources 2014, 256, 1–7.

[84] C. S. Yoon , D. W. Jun , S. T. Myung , Y. K. Sun , ACS Energy Lett. 2017, 2, 1150–1155.

[85] W. Wang , J. Zhang , D. Y. W. Yu , Q. Li , J. Power Sources 2017, 364, 420–425.

[86] J. Zhou, J. Lian, L. Hou, J. Zhang, H. Gou, M. Xia, Y. Zhao, T. A. Strobel, L. Tao, F. Gao, *Nat. Commun*. **2015**, *6*, 10.1038/ncomms9503. PMC4598840 26415838

[87] K. Leng , Z. Chen , X. Zhao , W. Tang , B. Tian , C. T. Nai , W. Zhou , K. P. Loh , ACS Nano 2016, 10, 9208–9215. 27636565 10.1021/acsnano.6b05746

[88] S. A. Siddiqui , A. Prado-Roller , H. Shiozawa , Mater. Adv. 2022, 3, 224–231. 35128414 10.1039/d1ma00866h PMC8724791

[89] H. K. Kim , W. S. Yun , M. B. Kim , J. Y. Kim , Y. S. Bae , J. D. Lee , N. C. Jeong , J. Am. Chem. Soc. 2015, 137, 10009–10015. 26197386 10.1021/jacs.5b06637

[90] C. Prestipino , L. Regli , J. G. Vitillo , F. Bonino , A. Damin , C. Lamberti , A. Zecchina , P. L. Solari , K. O. Kongshaug , S. Bordiga , Chem. Mater. 2006, 18, 1337–1346.

[91] H. S. Bhardwaj , T. Ramireddy , A. Pradeep , M. K. Jangid , V. Srihari , H. K. Poswal , A. Mukhopadhyay , ChemElectroChem 2018, 5, 1219–1229.

[92] K. Mazloomian , H. J. Lancaster , C. A. Howard , P. R. Shearing , T. S. Miller , Batteries Supercaps 2023, 6, e202300214.

[93] H. Zhong , K. H. Ly , M. Wang , Y. Krupskaya , X. Han , J. Zhang , J. Zhang , V. Kataev , B. Büchner , I. M. Weidinger , S. Kaskel , P. Liu , M. Chen , R. Dong , X. Feng , Angew. Chem. 2019, 131, 10787–10792. 10.1002/anie.201907002 31169942

[94] Q. Xia , S. Zhang , Y. Zhang , R. Bai , S. Li , J. Zhang , X. Chen , ACS Appl. Nano Mater. 2021, 4, 12108–12118.

[95] J. Kaur , A. Khanna , R. Kumar , R. Chandra , J. Mater. Sci. Mater. Electron. 2022, 33, 16154–16166.

[96] M. Amores , K. Wada , K. Sakaushi , H. Nishihara , J. Phys. Chem. C 2020, 124, 9215–9224.

[97] Z. Sang , Y. Tong , F. Hou , J. Liang , Trans. Tianjin Univ. 2023, 29, 136–150.

[98] A. J. Bard , L. R. Faulkner , H. S. White , in Electrochemical Methods: Fundamentals and Applications, John Wiley & Sons, Hoboken, NJ 2022.

[99] A. Noori , M. F. El-Kady , M. S. Rahmanifar , R. B. Kaner , M. F. Mousavi , Chem. Soc. Rev. 2019, 48, 1272–1341. 30741286 10.1039/c8cs00581h

[100] Y. Shao , M. F. El-Kady , J. Sun , Y. Li , Q. Zhang , M. Zhu , H. Wang , B. Dunn , R. B. Kaner , Chem. Rev. 2018, 118, 9233–9280. 30204424 10.1021/acs.chemrev.8b00252

[101] G. Vignesh , R. Ranjithkumar , P. Devendran , N. Nallamuthu , S. Sudhahar , M. Krishna Kumar , Mater. Sci. Eng., B 2023, 290, 116328.

[102] P. Salarizadeh , S. Azizi , H. Beydaghi , A. Bagheri , M. B. Askari , Molecules 2023, 28, 4613. 37375168 10.3390/molecules28124613 PMC10304452

[103] C. Portet , P. L. Taberna , P. Simon , C. Laberty-Robert , Electrochim. Acta 2004, 49, 905–912.

[104] C. Zhong , Y. Deng , W. Hu , J. Qiao , L. Zhang , J. Zhang , Chem. Soc. Rev. 2015, 44, 7484–7539. 26050756 10.1039/c5cs00303b

[105] F. Hadji , M. Omari , M. Mebarki , N. Gabouze , A. Layadi , J. Alloys Compd. 2023, 942, 169047.

[106] K. Qin , J. Baucom , L. Diao , Y. Lu , N. Zhao , Small 2022, 18, 1–9. 10.1002/smll.202203166 35871547

[107] X. Zhao , H. Li , M. Zhang , W. Pan , Z. Luo , X. Sun , ACS Appl. Mater. Interfaces 2022, 14, 34781–34792. 35867900 10.1021/acsami.2c08903

[108] A. Jesús Cortés De La Torre , J. A. Ávila-Niño , R. Antaño-López , E. Araujo , ACS Appl. Nano Mater. 2022, 5, 15700–15710.

[109] M. R. Biradar , A. V. Salkar , P. P. Morajkar , S. V. Bhosale , S. V. Bhosale , J. Storage Mater. 2022, 48, 103953.

[110] V. S. Kumbhar , D. H. Kim , Electrochim. Acta 2018, 271, 284–296.

[111] D. K. Ngyuen , I. M. Schepisi , F. Z. Amir , Chem. Eng. J. 2019, 378, 122150.

[112] S. A. Beknalkar , A. M. Teli , N. S. Harale , D. S. Patil , S. A. Pawar , J. C. Shin , P. S. Patil , Appl. Surf. Sci. 2021, 546, 149102.

[113] N. R. Chodankar , P. A. Shinde , S. J. Patil , S. K. Hwang , G. S. R. Raju , K. S. Ranjith , D. P. Dubal , Y. S. Huh , Y. K. Han , Energy Storage Mater. 2021, 39, 194–202.

[114] N. R. Chodankar , H. D. Pham , A. K. Nanjundan , J. F. S. Fernando , K. Jayaramulu , D. Golberg , Y. K. Han , D. P. Dubal , Small 2020, 16, 2002806. 10.1002/smll.202002806 32761793

[115] K. Jayaramulu , M. Horn , A. Schneemann , H. Saini , A. Bakandritsos , V. Ranc , M. Petr , V. Stavila , C. Narayana , B. Scheibe , Š. Kment , M. Otyepka , N. Motta , D. Dubal , R. Zbořil , R. A. Fischer , Adv. Mater. 2021, 33, 2004560. 33274794 10.1002/adma.202004560 PMC11468759

[116] Z. Li , H. Li , J. Song , T. Liu , Y. He , A. Meng , Y. Liu , C. Chen , C. Sun , M. Hu , L. Wang , G. Li , J. Zhao , Energy Storage Mater. 2022, 50, 252–264.

[117] X. Xin , Y. Xu , H. Wuliji , F. Sun , Q. Liu , Z. Wang , T. R. Wei , X. Zhao , X. Song , L. Gao , ACS Nano 2023, 17, 657–667. 36542067 10.1021/acsnano.2c09970

[118] M. Eredia , S. Bellani , M. I. Zappia , L. Gabatel , V. Galli , A. Bagheri , H. Beydaghi , G. Bianca , I. Conticello , V. Pellegrini , F. Bonaccorso , APL Mater. 2022, 10, 101102.

[119] J. Huang , Y. Xiong , Z. Peng , L. Chen , L. Wang , Y. Xu , L. Tan , K. Yuan , Y. Chen , ACS Nano 2020, 14, 14201–14211. 33012161 10.1021/acsnano.0c07326

[120] X. Wei , B. Qiu , L. Xu , Q. Qin , W. Zhang , Z. Liu , F. Wei , Y. Lv , J. Storage Mater. 2023, 62, 1–10.

[121] Y. Wu , X. Jia , H. Zhang , F. Zhou , Z. Fu , X. Jia , Z. Li , F. Liu , L. Wang , Z. Xiao , J. Storage Mater. 2023, 62, 106855.

[122] S. Ban , J. Zhang , L. Zhang , K. Tsay , D. Song , X. Zou , Electrochim. Acta 2013, 90, 542–549.

[123] M. Kim , C. Wang , J. Earnshaw , T. Park , N. Amirilian , A. Ashok , J. Na , M. Han , A. E. Rowan , J. Li , J. W. Yi , Y. Yamauchi , J. Mater. Chem. A 2022, 10, 24056–24063.

[124] L. G. Beka , X. Bu , X. Li , X. Wang , C. Han , W. Liu , RSC Adv. 2019, 9, 36123–36135. 35540587 10.1039/c9ra07061c PMC9074924

