# Supplementary material for: Coexistence of Redox‐Active Metal and Ligand Sites in Copper‐Based Two‐Dimensional Conjugated Metal–Organic Frameworks as Active Materials for Battery‐Supercapacitor Hybrid Systems
Source: ChemSusChem. 2024 Dec 19;18(4):e202401454. doi: 10.1002/cssc.202401454 (PMC11826127; doi:10.1002/cssc.202401454)
Supplement: Supplementary file 1 — Supporting Information [file CSSC-18-e202401454-s001.pdf]

ChemSusChem

Supporting Information

Coexistence of Redox-Active Metal and Ligand Sites in Copper-Based Two-Dimensional Conjugated Metal–Organic Frameworks as Active Materials for Battery-Supercapacitor Hybrid Systems

Ahmad Bagheri, Sebastiano Bellani,* Hossein Beydaghi, Zhiyong Wang, Ahiud Morag, Marilena I. Zappia, Jaya-Kumar Panda, Samaneh Vaez, Valentina Mastronardi, Agnese Gamberini, Sanjay Balkrishna Thorat, Matteo Abruzzese, Lea Pasquale, Renhao Dong, Minghao Yu, Xinliang Feng, and Francesco Bonaccorso*

Supporting information

Coexistence of Redox-Active Metal and Ligand Sites in Copper-based Two-Dimensional Conjugated Metal-Organic Frameworks as Active Materials for Battery-Supercapacitor Hybrid Systems

Ahmad Bagheri,[a,b,c] Sebastiano Bellani,[c] Hossein Beydaghi,[c] Zhiyong Wang,[b,d] Ahiud Morag,[b,d] Marilena I. Zappia,[c] Jaya-Kumar Panda,[c] Samaneh Vaez,[c,e] Valentina Mastronardi,[c] Agnese Gamberini,[c] Sanjay Balkrishna Thorat,[c] Matteo Abruzzese,[c] Lea Pasquale,[f] Renhao Dong,[b,d] Minghao Yu,[b] Xinliang Feng,[b,d] and Francesco Bonaccorso [a,c*]*

[a] Graphene Labs, Istituto Italiano di Tecnologia, via Morego 30, 16163 Genoa, Italy

[b] Center for Advancing Electronics Dresden (cfaed) & Faculty of Chemistry and Food Chemistry, Technische Universität Dresden, 01062 Dresden, Germany

[c] BeDimensional S.p.A., Lungotorrente Secca 30R, 16163 Genoa, Italy

[d] Max Planck Institute of Microstructure Physics, Weinberg 2, 06120 Halle, Germany

[e] Department of Applied Science and Technology (DISAT), Politecnico di Torino, 10129 Torino, Italy

[f] Materials Characterization Facility, Istituto Italiano di Tecnologia, Via Morego 30, 16163 Genova, Italy

Keywords

Metal-organic frameworks (MOFs), batteries, supercapacitors, energy storage, redox-active sites.

Experimental methods

Materials

Sodium nitrate ($NaNO_3$), polyvinylidene fluoride (PVDF), 1-methyl-2-pyrrolidone (NMP), Ag wire (diam. 1.5 mm, ≥99.99% trace metals basis), copper(II) acetate ($Cu(OAc)_2$), NaOH, Whatman glass fiber filter, sodium dodecyl sulfate (SDS), acetone and ethanol were purchased from Sigma Aldrich. Tetrahydroxy-1,4-quinone (THQ) was purchased from Fluorochem. 1 M lithium hexafluorophosphate ($LiPF_6$) in ethylene carbonate (EC) and dimethyl carbonate (DMC) (1:1 vol/vol), activated carbon (AC) powder (AB520Y), and carbon black (Super-P) were purchased from Solvionic, MTI corporation, and Alfa Aesar, respectively. All the chemicals were used as received without any further purification.

Synthesis of porous hexahydroxybenzene-Cu (HHB-Cu) nanosheets[1]

In a 500 mL conical flask, a solution was prepared by combining $Cu(OAc)_2$ (480 mg, 2.61 mmol) and SDS (150 mg, 0.51 mmol) in 150 mL of water. Afterwards, a solution of NaOH (150 mg, 3.75 mmol) in 150 mL of water was added to the $Cu(OAc)_2$ and SDS solution, along with THQ (300 mg, 1.74 mmol) powder. The resulting mixture was subjected to sonication at 50°C for 30 min, followed by a rest period of 10 h at 25°C. The resulting precipitate was collected, washed with water and ethanol in an ultrasonic ice bath for 30 min, and allowed to settle for 24 h to precipitate the largest particles. The upper colloidal suspension of HHB-Cu nanosheets was collected and dried under a vacuum at 100°C for 12 h. This process was repeated until the particles were fully exfoliated, resulting in HHB-Cu nanosheets with a yield of 87.5%. The reaction can be upscaled by proportionally increasing the monomers, SDS, and base in the mixture.

Synthesis of non-porous HHB-Cu nanosheets

A reaction container in the form of a 500 mL conical flask was utilized. To prepare the reaction mixture, $Cu(OAc)_2$ (480 mg, 2.61 mmol) and SDS (150 mg, 0.51 mmol) were dissolved in 300 mL of water. Subsequently, THQ (300 mg, 1.74 mmol) powder was added to the mixture. The resulting mixture was then subjected to sonication at 50°C for 30 min, followed by allowing the reaction system to remain undisturbed for 10 h at 80°C. The precipitate formed was collected and washed with water, ethanol, and acetone. Afterward, it was dried under vacuum conditions at

100°C for 12 h, resulting in a 75.5% isolated yield. It should be noted that this reaction can be upscaled by proportionally increasing the quantities of monomers and SDS in the mixture.

Preparation of electrodes based on HHB-Cu nanosheets

HHB-Cu or np-HHB-Cu powders were mixed with carbon black (Super-P) and PVDF in a material mass ratio of 80:10:10. The mixture was then thoroughly ground in a mortar using NMP as the solvent for 30 min. The resulting uniform slurry was cast onto small discs (8 mm diameter) of graphite paper, which served as the current collector, and dried at room temperature. The electrodes were further dried in a vacuum oven at 80°C for 8 h before assembling them in Swagelok cells. The electrode mass loadings (including conductive additives and binders) were between 1.5 and 2.0 mg cm^{-2}.

Preparation of the AC-based counter electrodes.

Activated carbon powders were mixed with carbon black (Super-P) and PVDF in a material weight ratio of 80:10:10, using NMP as the solvent. The mixture was cast onto graphite paper discs punched with a diameter of 10 mm and dried overnight at 120°C in a vacuum oven. The electrode mass loadings were between 20 and 30 mg cm^{-2}.

Standard three-electrode cell assembly.

In a Swagelok cell, the HHB-Cu-based electrodes served as the working electrode, while AC-based electrodes acted as counter electrode with over-sized capacitance. In 1 M $LiPF_6$ in EC/DMC, Ag wire was used as the pseudo-reference electrode, whereas Ag/AgCl was used as the reference electrode in 10 M $NaNO_3$ aqueous electrolyte. A glass microfiber filter membrane (Whatman, GF/D) was used as the separator. For organic electrolytes, all cells were assembled under dry and oxygen-free conditions in an Ar-filled glove box.

Material and device characterization.

Transmission electron microscopy (TEM, Zeiss, Libra 200 KV) and scanning electron microscopy (SEM, Zeiss Gemini 500) were utilized to examine the morphology and structure of the samples. For SEM analysis, the synthetic nanosheets of HHB-Cu were deposited on Si substrates, while for TEM characterizations, Cu grids were used. An aberration-corrected high-resolution transmission electron microscopy (AC-HRTEM) imaging technique was employed using a 300 kV FEI Titan 80-300 microscope with image-side Cs-correction. The specific surface area was determined by applying the Brunauer-Emmett-Teller (BET) method at 77 K under low-pressure conditions. The X-ray diffraction (XRD) analysis of the samples was performed using a PANalytical Empyrean

X-ray diffractometer with Cu Kα radiation. Raman spectroscopy analysis was conducted at an exciting wavelength of 532 nm using a Renishaw micro-Raman Invia 1000 spectrophotometer. In both XRD and Raman analyses, the samples were prepared by drop casting a diluted dispersion of the materials onto Si/SiO_2 substrates and subsequently dried under vacuum for 12 h.

Electrochemical characterization and calculations.

The electrochemical characterization of the supercapacitors involved cyclic voltammetry (CV), galvanostatic charge/discharge (GCD), and electrochemical impedance spectroscopy (EIS) measurements. These measurements were conducted at room temperature (25 °C) using a potentiostat/galvanostat (VMP3, Biologic). Cyclic voltammetry measurements were performed at different potential scan rates ranging from 0.5 mV s^{-1} to 50 mV s^{-1}. Galvanostatic charge/discharge measurements were carried out at various specific currents, ranging from 0.02 to 10 A g^{-1}. The specific capacity (C_s, mAh g^{-1}) of the electrodes were calculated from GCD curves using the equation (S1):

$$C_s = \frac{|i| \times t_d}{m} \times \frac{1000}{3600} \qquad \text{(S1)}$$

where m is the mass loading (mg) of the electrode (excluding the current collector), i is the charging/discharging current (mA), t_d is the discharge time of the GCD curve (s).

Fabrication of Hybrid supercapacitors (HSCs).

Hybrid supercapacitors were constructed in Swagelok cells, where the HHB-Cu-based negative electrode (*i.e.,* p-HHB-Cu, and np-HHB-Cu) and the AC positive electrode were separated by a glass fiber membrane. Both organic and aqueous electrolytes were screened. The C_s of the electrodes were balanced according to the following equation:

$$C_{+} \times m_{+} = C_{-} \times m_{-} \qquad \text{(S2)}$$

where C_+ and C_+ are the C_s of the positive and negative electrodes respectively, while m_+ and m_- are the masses of positive and negative electrodes (excluding the masses of their current collectors). The C_s of HSCs were calculated according to Equation S1, but considering m as the mass of the materials of both electrodes (excluding current collectors). The HSCs with organic electrolytes were assembled under dry and oxygen-free conditions in an Ar-filled glove box.

The Es (Wh kg^{-1}) and Ps (W kg^{-1}) of the devices were calculated using the following equations:

$$Es\ (\mathrm{Wh\ kg^{-1}}) = \frac{i_d}{3.6 \times M} \int V dt \qquad \text{(S3)}$$

$$Ps\ (\mathrm{W\ kg^{-1}}) = 3600 \times \frac{Ed}{t_d} \qquad \text{(S4)}$$

where ∫Vdt is the area under the galvanostatic discharge curve, i_d is the specific current (A g^{-1}), m M is the mass of the materials of both electrodes (excluding current collectors) and E_d is the discharge specific energy. Electrochemical impedance spectroscopy measurements of the HSCs were performed in the frequency range from 0.01 Hz to 200 kHz at the discharged state with an AC voltage amplitude of 20 mV.

The Coulombic efficiency (CE) of the HSCs was calculated by the ratio of the t_d and charge time (t_c) of the GCD curve, *i.e.*;

$$CE = t_d/t_c \qquad (S5)$$

Table S1. Comparative BET and conductivity analysis for p-HHB-Cu and np-HHB-Cu.

	**SSA (BET method, m^2g^{-1})**	**Conductivity (Van der Pauw method, S cm^{-1})**
p-HHB-Cu $Cu_3(C_6O_6)_2$	385	1.53×10^{-7}
np-HHB-Cu $Cu_3(C_6O_6)$	-	2.58×10^{-2}

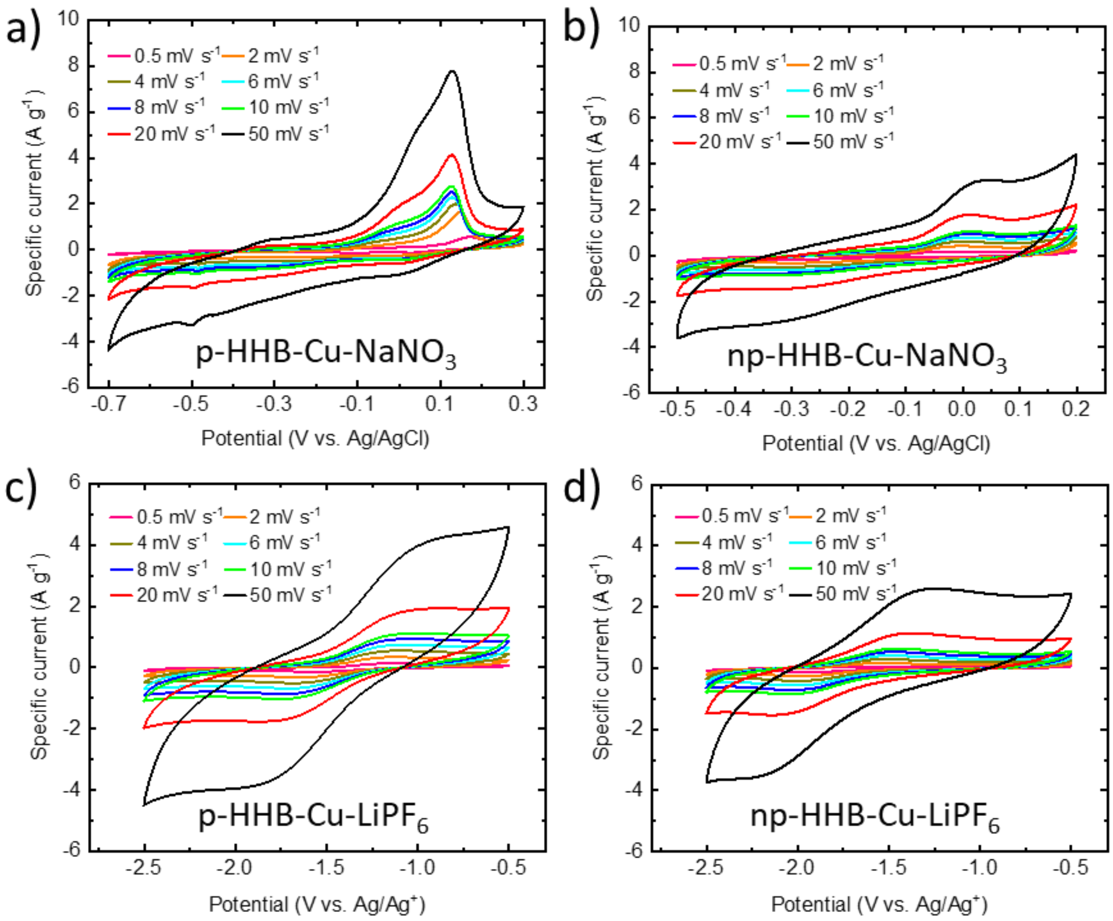

Figure S1. *Electrochemical characterization of the investigated HHB-Cu-based electrodes. CV curves measured for a) p-HHB-Cu-$NaNO_3$, b) np-HHB-Cu-$NaNO_3$, c) p-HHB-Cu-$LiPF_6$, and d) np-HHB-Cu-$LiPF_6$, at potential scan rates ranging from 0.5 to 50 mV s^{-1}.*

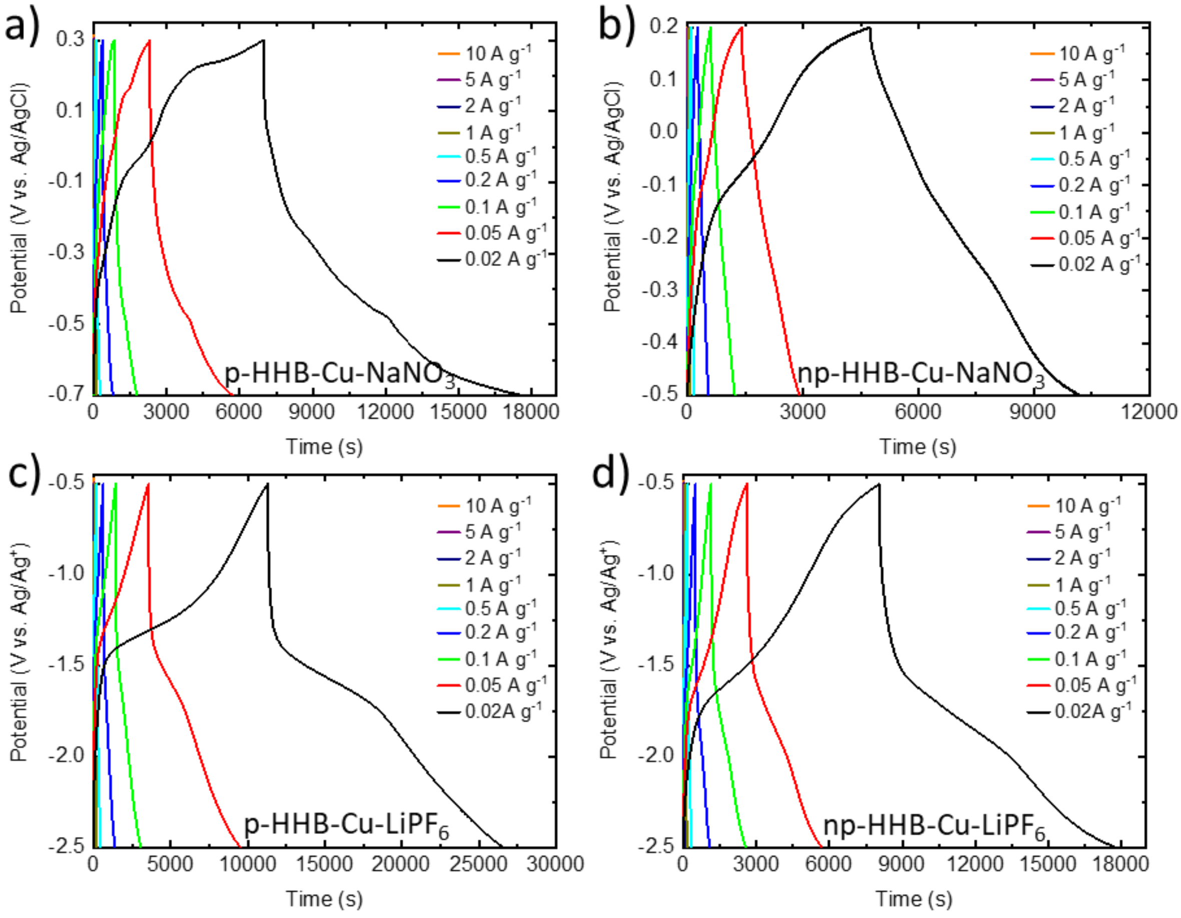

Figure S2. *Electrochemical characterization of the investigated HHB-Cu-based electrodes. GCD profiles measured for a) p-HHB-Cu-$NaNO_3$, b) np-HHB-Cu-$NaNO_3$, c) p-HHB-Cu-$LiPF_6$, and d) np-HHB-Cu-$LiPF_6$, at specific currents ranging from 0.02 to 10 A g^{-1}.*

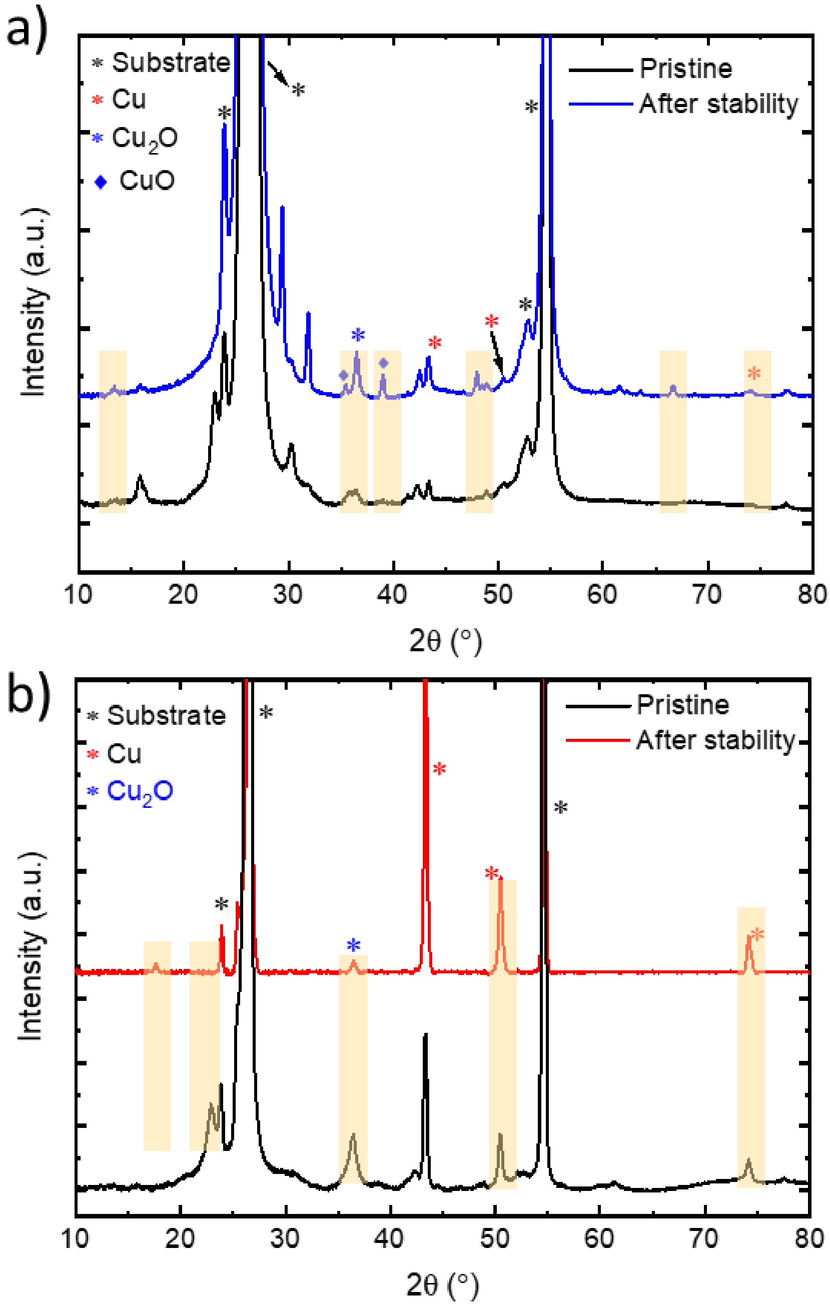

Figure S3. XRD patterns measured for a) p-HHB-Cu-$NaNO_3$, and b) np-HHB-Cu-$NaNO_3$ electrodes before and after 3000 GCD cycles at 1 A g^{-1} in 10 M $NaNO_3$.

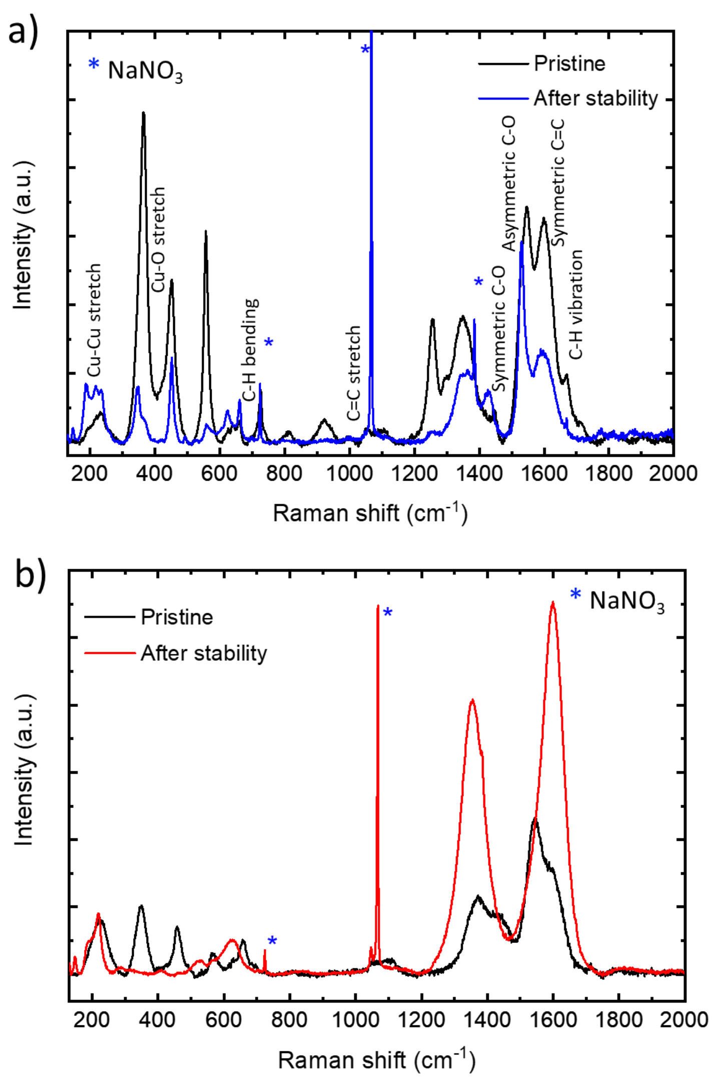

Figure S4. Raman spectra measured for a) p-HHB-Cu-NaNO₃, and b) np-HHB-Cu-NaNO₃ electrodes before and after 3000 GCD cycles at 1 A g^{-1} in 10 M NaNO₃.

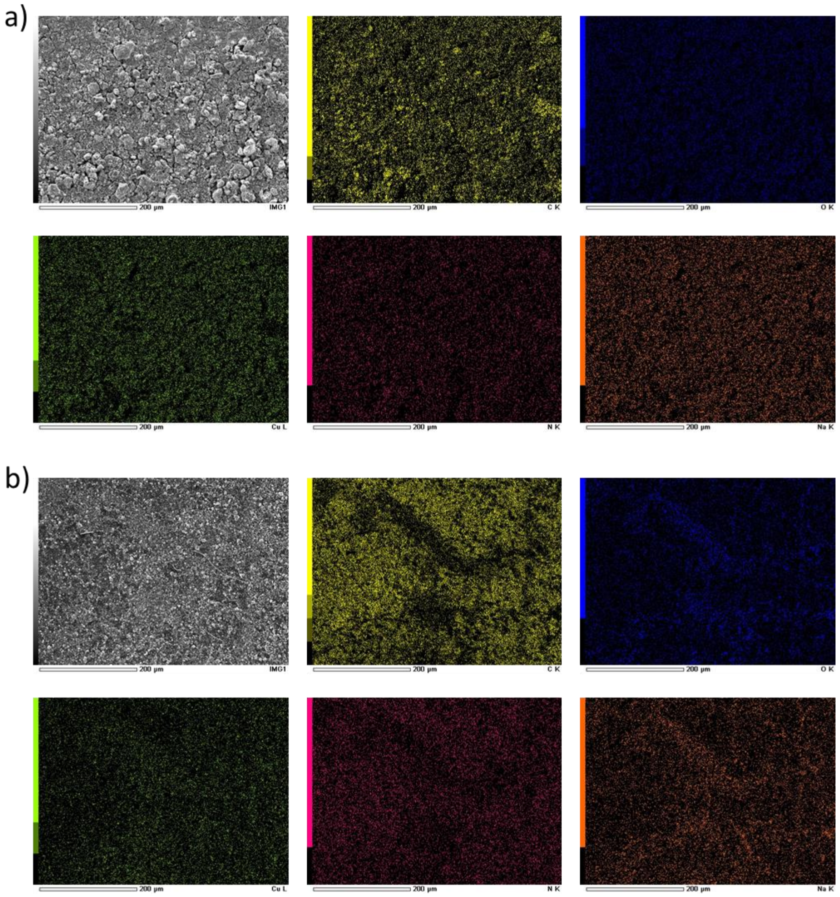

Figure S5. SEM imaging and elemental mapping of p-HHB-Cu-$NaNO_3$ electrodes a) before and b) after 3000 GCD cycles at 1 A g^{-1} in 10 M $NaNO_3$.

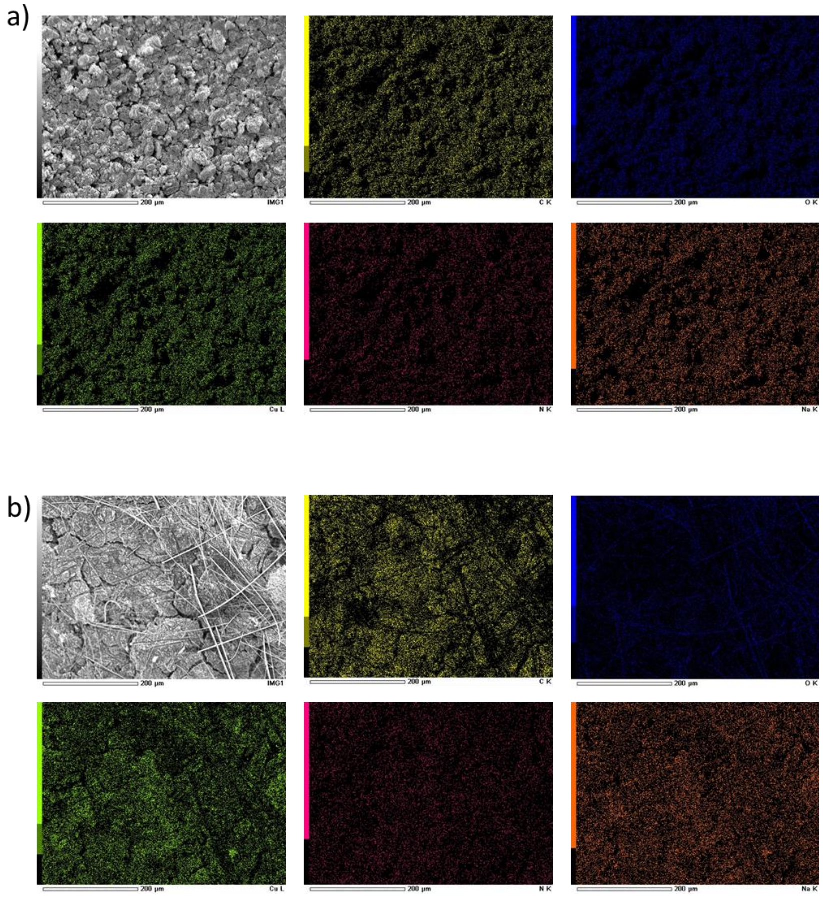

Figure S6. SEM imaging and elemental mapping of np-HHB-Cu-$NaNO_3$ electrodes a) before and b) after 3000 GCD cycles at 1 A g^{-1} in 10 M $NaNO_3$.

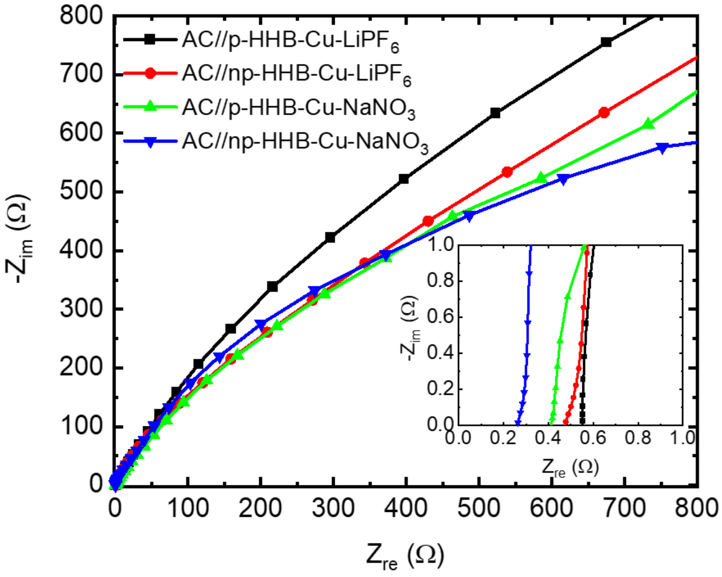

Figure S7. Nyquist plots measured for different HHB-Cu-based electrodes in a three-electrode cell configuration; the inset shows the magnified view of the high-frequency region.

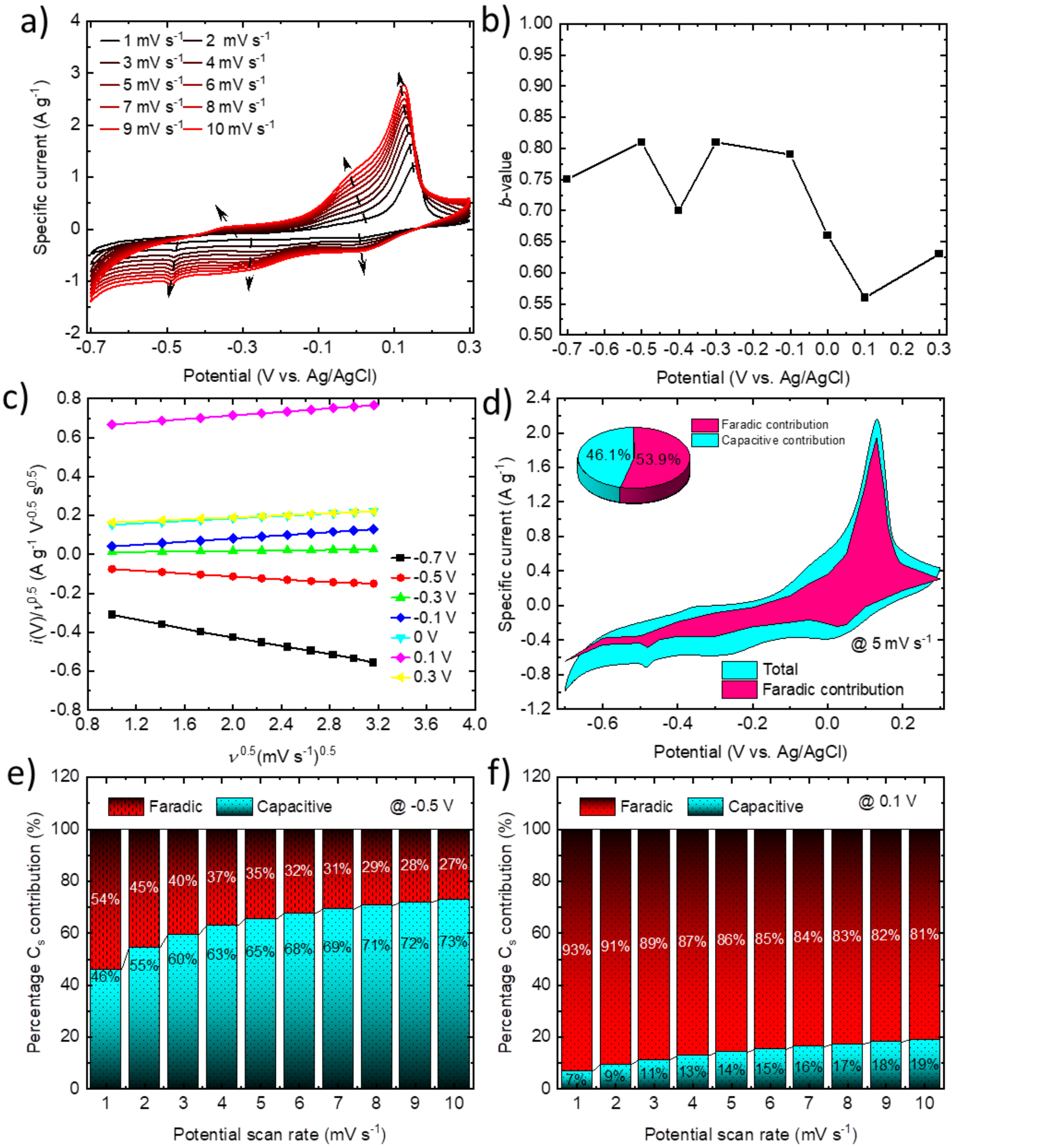

Figure S8. Charge-storage mechanism of p-HHB-Cu-NaNO$_3$. a) CV curves measured at voltage scan rates ranging from 1 to 10 mV s^{-1} and b) corresponding b-values as a function of the potential. c) The linear relation of i(V)/v$^{0.5}$ vs. v$^{0.5}$ at various voltages. d) Capacitive and (Faradaic) diffusion-controlled currents determining the overall charge storage at 5 mV s^{-1}. Percentage Cs contribution of capacitive and Faradaic (diffusion-controlled) processes at e) -0.5 V and f) 0.1 V (vs. Ag/AgCl).

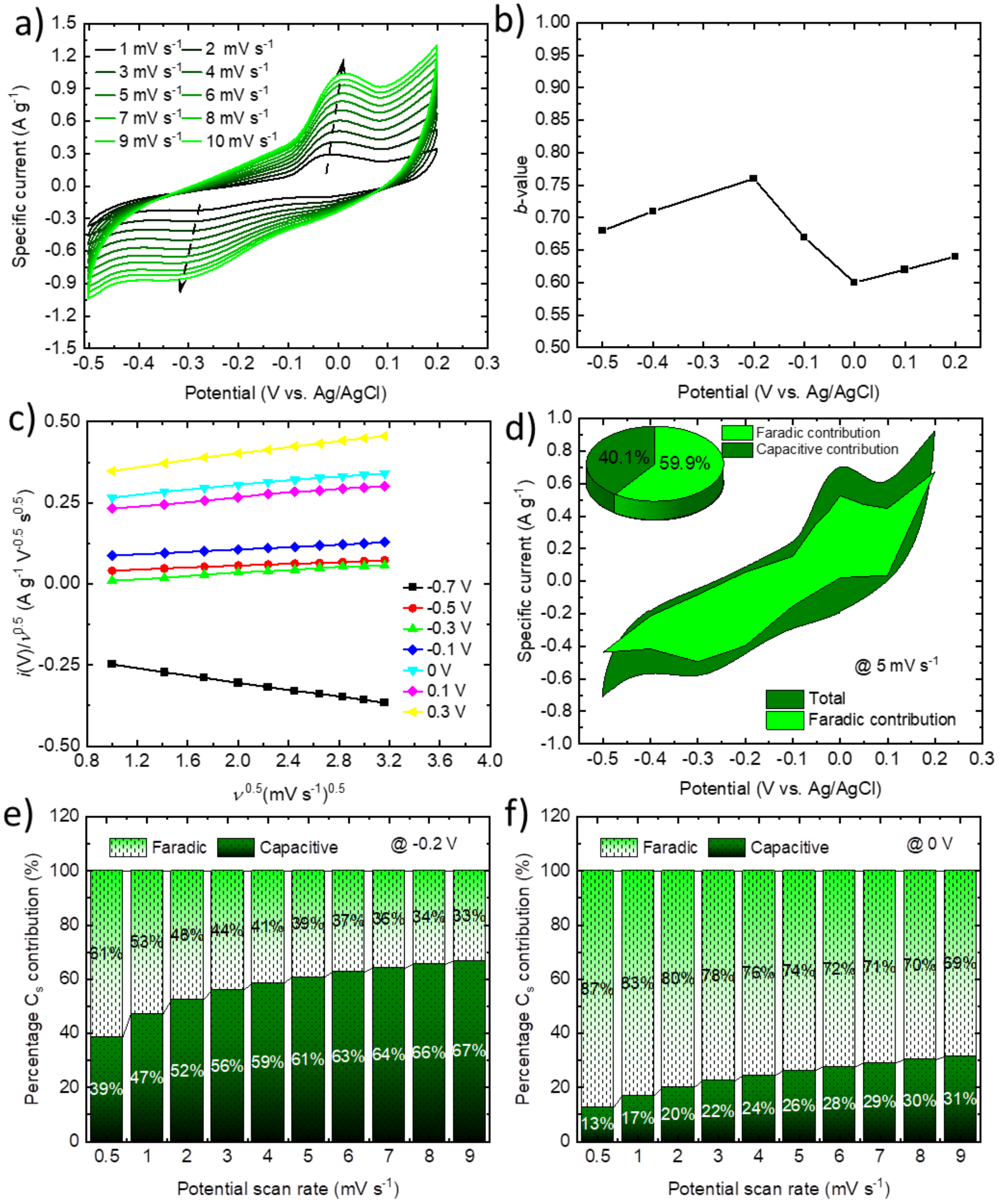

Figure S9. Charge-storage mechanism of np-HHB-Cu-NaNO$_3$ electrode. a) CV curves measured at voltage scan rates ranging from 1 to 10 mV s^{-1} and b) corresponding b-values as a function of the potential. c) The linear relation of i(V)/v$^{0.5}$ vs. v$^{0.5}$ at various voltages. d) Capacitive and (Faradaic) diffusion-controlled currents determining the overall charge storage at 5 mV s^{-1}. Percentage Cs contribution of capacitive and Faradaic (diffusion-controlled) processes at e) -0.2 V and f) 0.0 V (vs. Ag/AgCl).

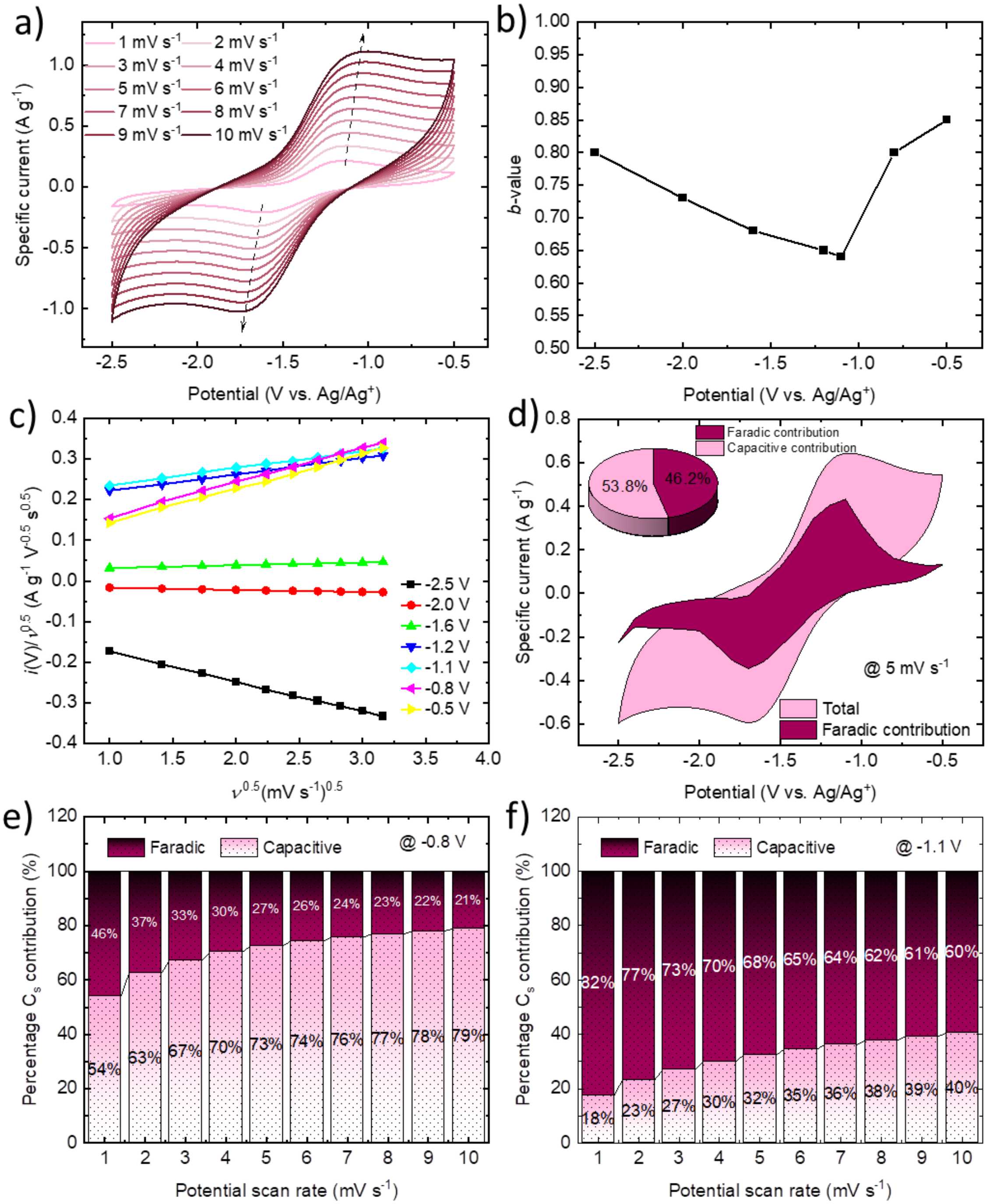

Figure S10. Charge-storage mechanism of p-HHB-Cu-LiPF$_6$ electrode. a) CV curves measured at voltage scan rates ranging from 1 to 10 mV s^{-1} and b) corresponding b-values as a function of the potential. c) The linear relation of i(V)/v$^{0.5}$ vs. v$^{0.5}$ at various voltages. d) Capacitive and (Faradaic) diffusion-controlled currents determining the overall charge storage at 5 mV s^{-1}. Percentage Cs contribution of capacitive and Faradaic (diffusion-controlled) processes at e) -0.8 V and f) -1.1 V (vs. Ag/Ag$^+$).

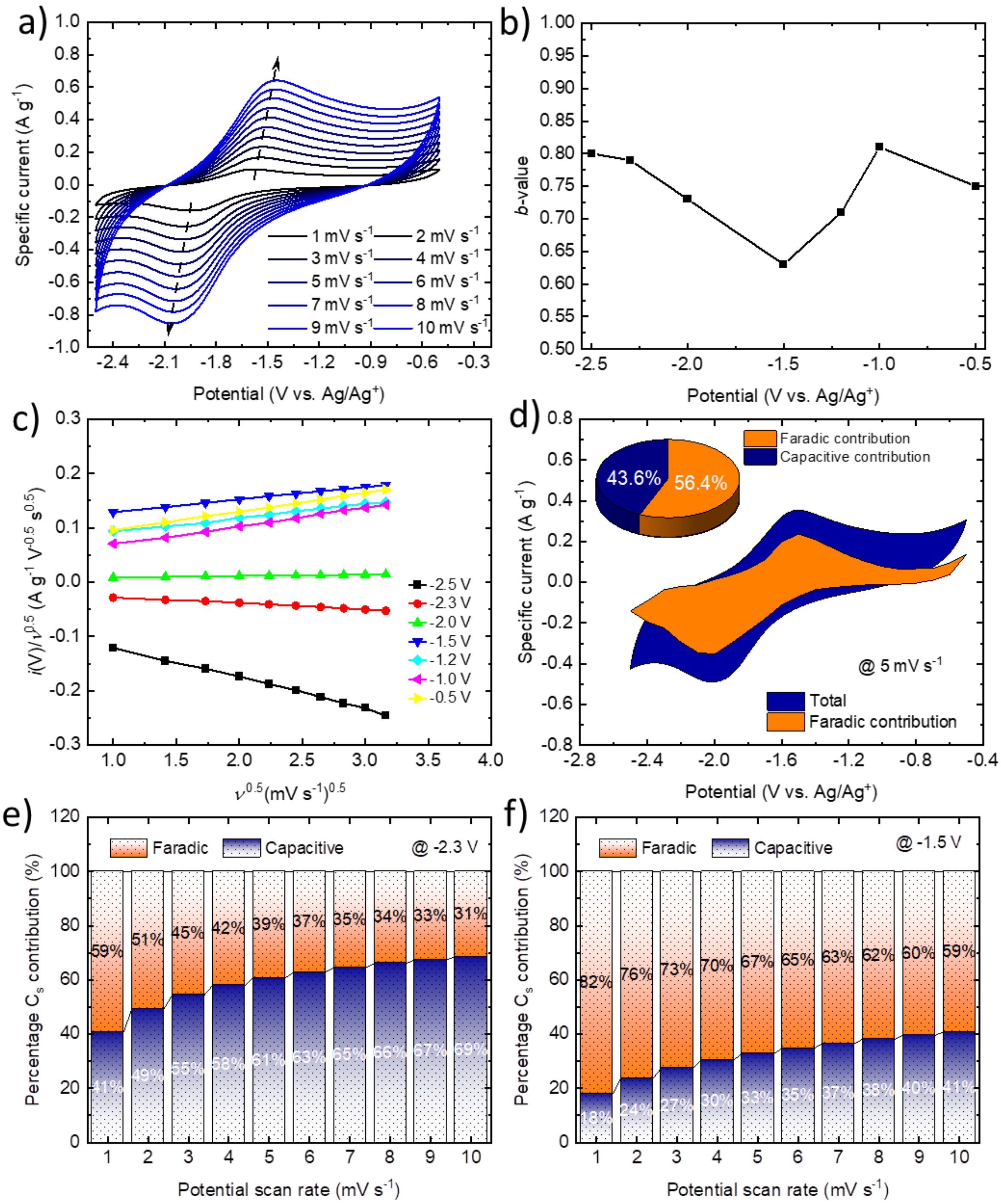

Figure S11. Charge-storage mechanism of np-HHB-Cu-LiPF$_6$ electrode. a) CV curves measured at voltage scan rates ranging from 1 to 10 mV s^{-1} and b) corresponding b-values as a function of the potential. c) The linear relation of $i(V)/\nu^{0.5}$ vs. $\nu^{0.5}$ at various voltages. d) Capacitive and (Faradaic) diffusion-controlled currents determining the overall charge storage at 5 mV s^{-1}. Percentage Cs contribution of capacitive and Faradaic (diffusion-controlled) processes at e) -2.3 V and f) -1.5 V (vs. Ag/Ag$^+$).

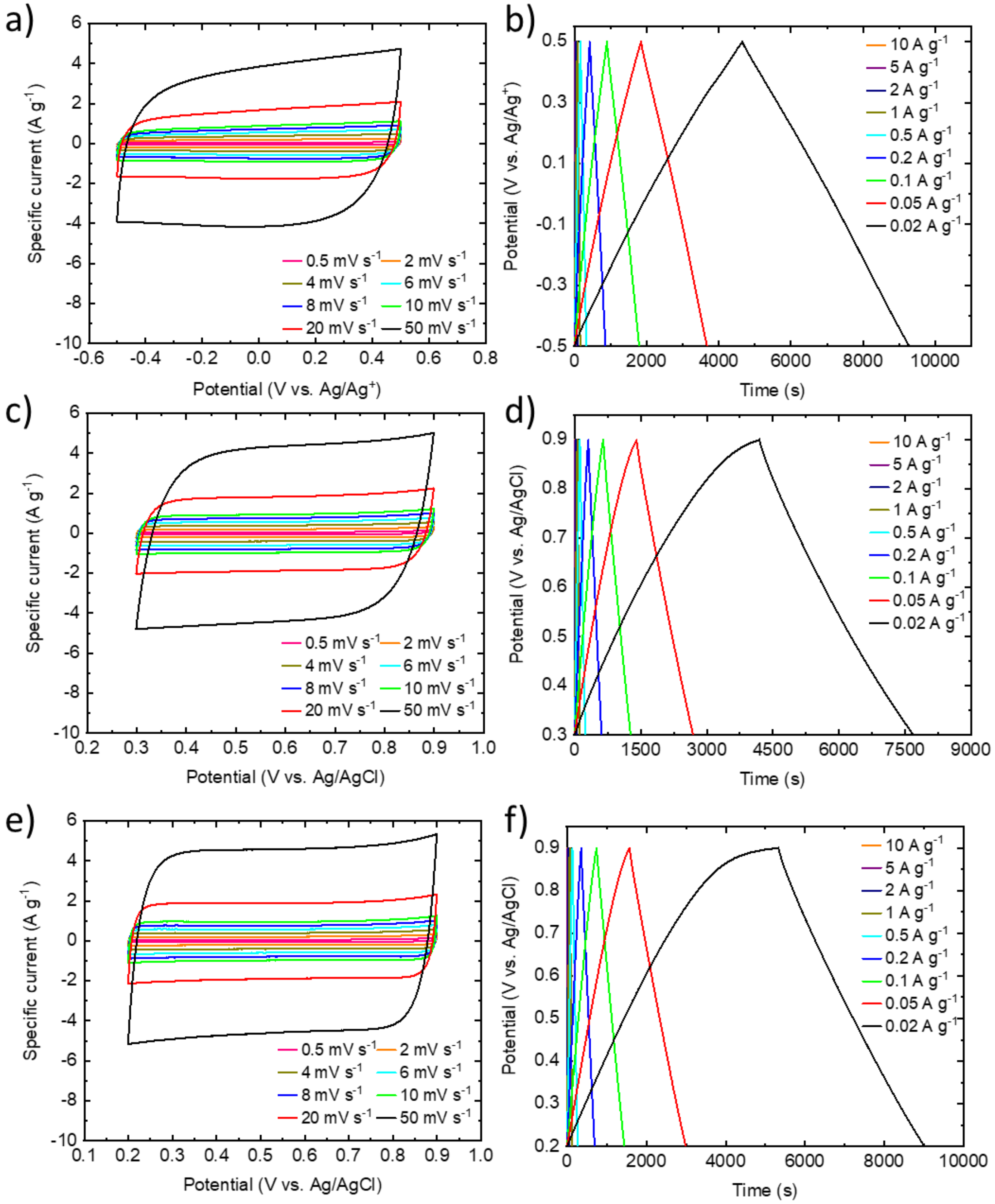

Figure S12. Electrochemical characterization of the investigated AC-based electrode, which was used as positive electrode in HSCs. a) CV curves and b) GCD profiles measured in 1 M $LiPF_6$ in EC/DMC from -0.5 to 0.5 V (vs. Ag/Ag^+), c) CV curves and d) GCD profiles measured in 10 M $NaNO_3$ from 0.2 to 0.9 V (vs. Ag/AgCl), e) CV curves and f) GCD profiles measured in 10 M $NaNO_3$ from 0.3 to 0.9 V (vs. Ag/AgCl).

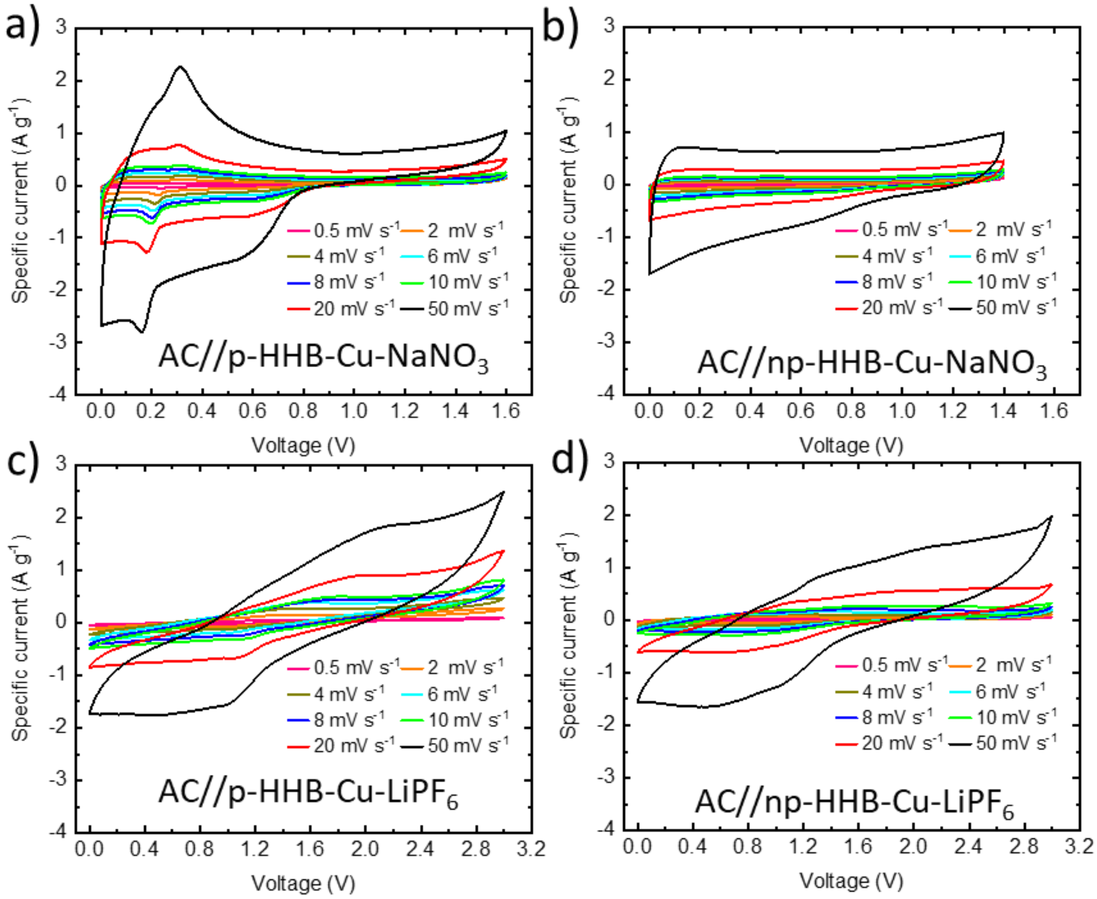

Figure S13. Electrochemical characterization of the investigated HSCs. CV curves measured for a) AC//p-HHB-Cu-NaNO$_3$, b) AC//np-HHB-Cu-NaNO$_3$, c) AC//p-HHB-Cu-LiPF$_6$, and d) AC//np-HHB-Cu-LiPF$_6$ at voltage scan rates ranging from 0.5 to 50 mV s^{-1}.

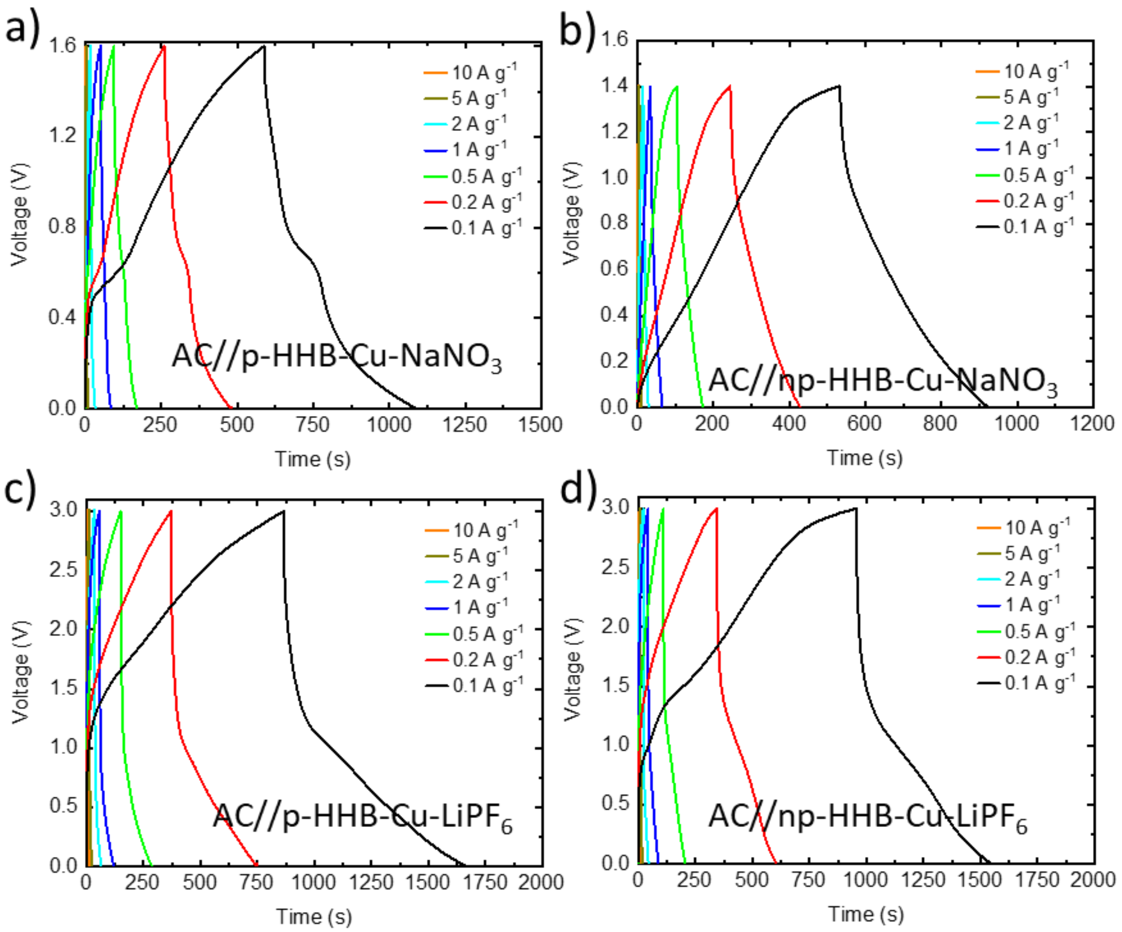

Figure S14. Electrochemical characterization of the investigated HSCs. GCD profiles measured for a) AC//p-HHB-Cu-$NaNO_3$, b) AC//np-HHB-Cu-$NaNO_3$, c) AC//p-HHB-Cu-$LiPF_6$, and d) AC//np-HHB-Cu-$LiPF_6$ a at specific currents ranging from 0.1 to 10 A g^{-1}

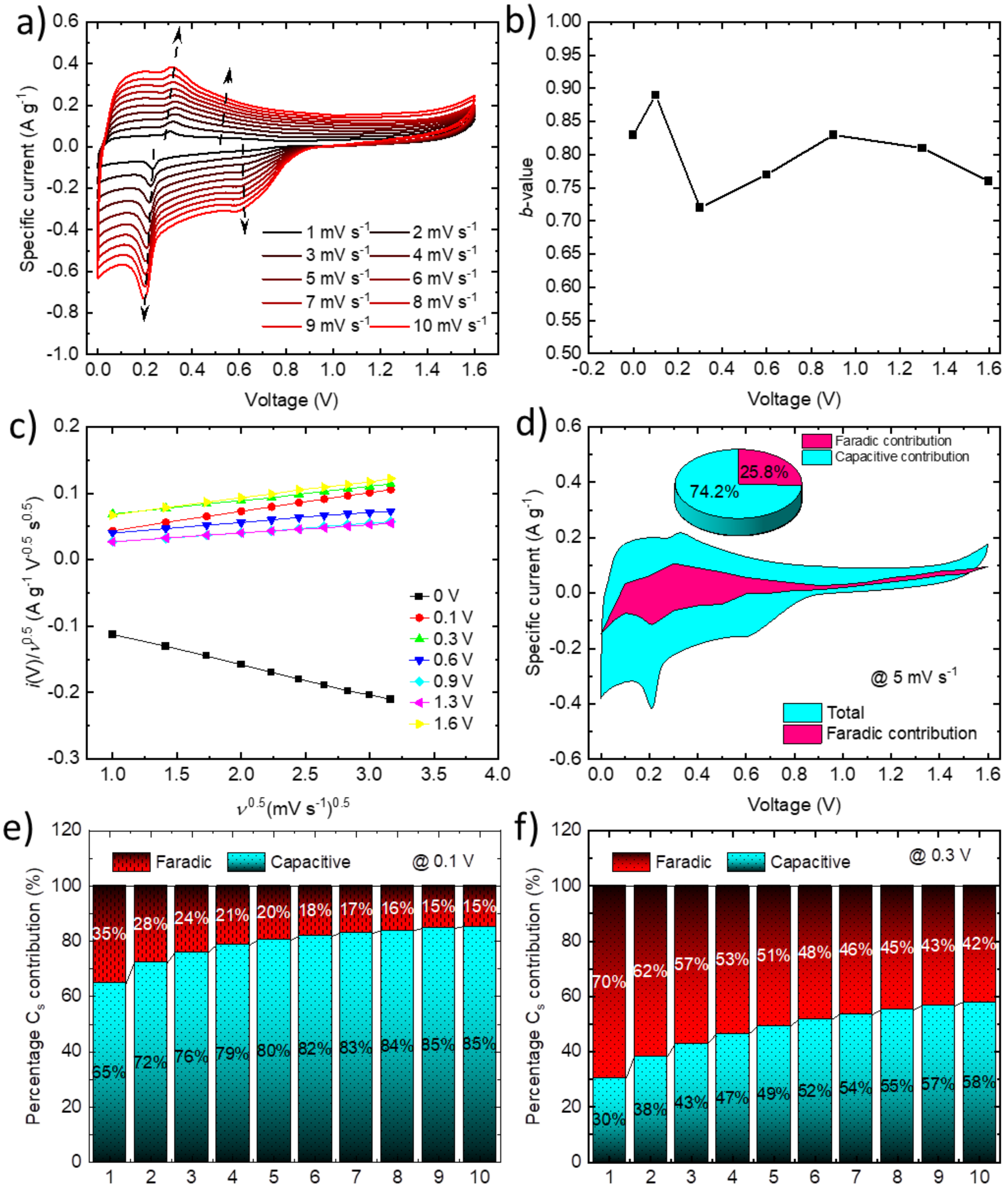

Figure S15. Charge storage mechanism of AC//p-HHB-Cu-$NaNO_3$ HSC. a) CV curves measured at voltage scan rates ranging from 1 to 10 mV s^{-1} and b) corresponding b-values as a function of the voltage. c) The linear relation of $i(V)/v^{0.5}$ vs. $v^{0.5}$ at various voltages. d) Capacitive and (Faradaic) diffusion-controlled currents determining the overall charge storage at 5 mV s^{-1}. Percentage Cs contribution of capacitive and Faradaic (diffusion-controlled) processes at e) 0.1 V and f) 0.3 V

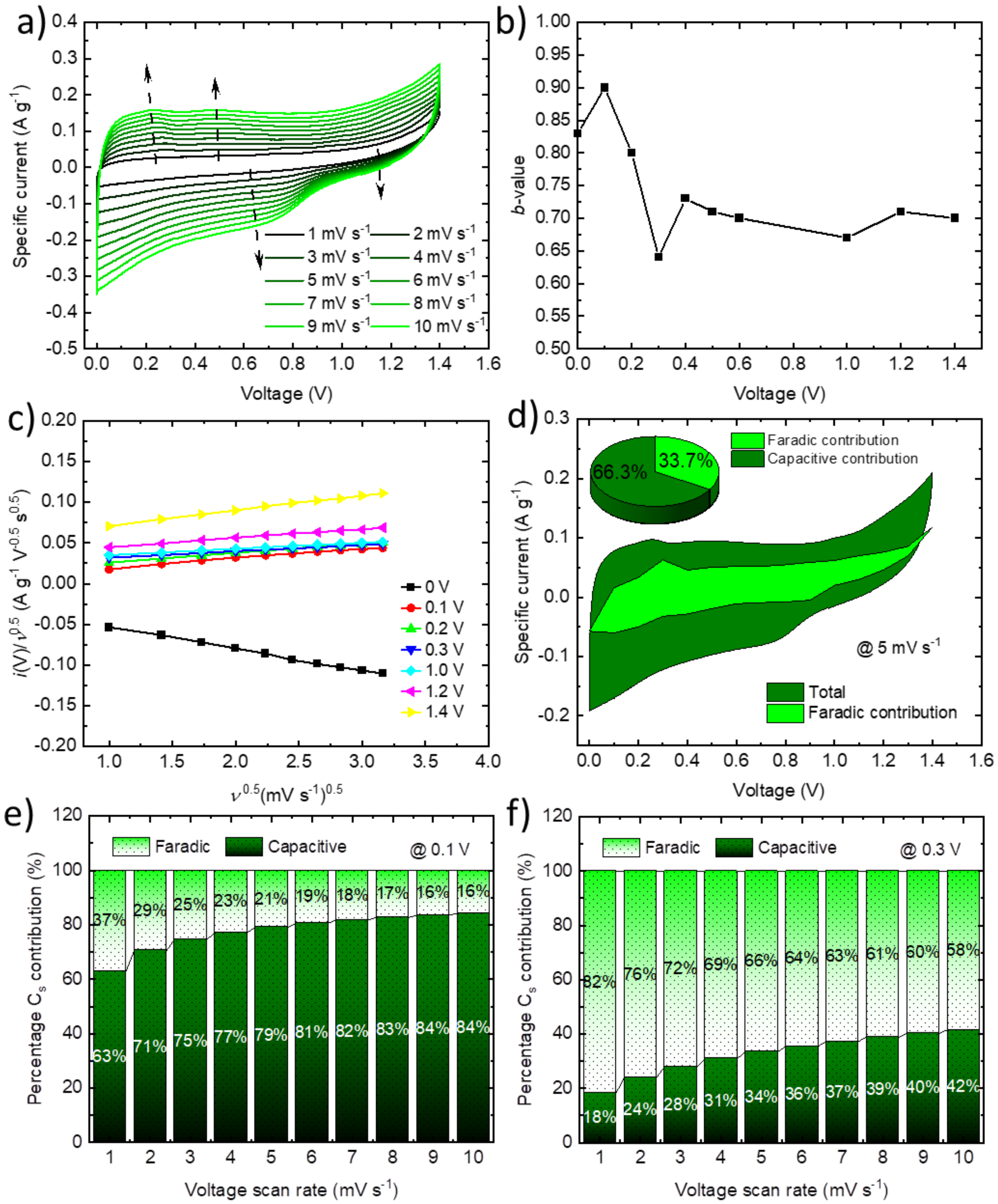

Figure S16. Charge storage mechanism of AC//np-HHB-Cu-$NaNO_3$ HSC. a) CV curves measured at voltage scan rates ranging from 1 to 10 mV s^{-1} and b) corresponding b-values as a function of the voltage. c) The linear relation of $i(V)/v^{0.5}$ vs. $v^{0.5}$ at various voltages. d) Capacitive and (Faradaic) diffusion-controlled currents determining the overall charge storage at 5 mV s^{-1}. Percentage Cs contribution of capacitive and Faradaic (diffusion-controlled) processes at e) 0.1 V and f) 0.3 V.

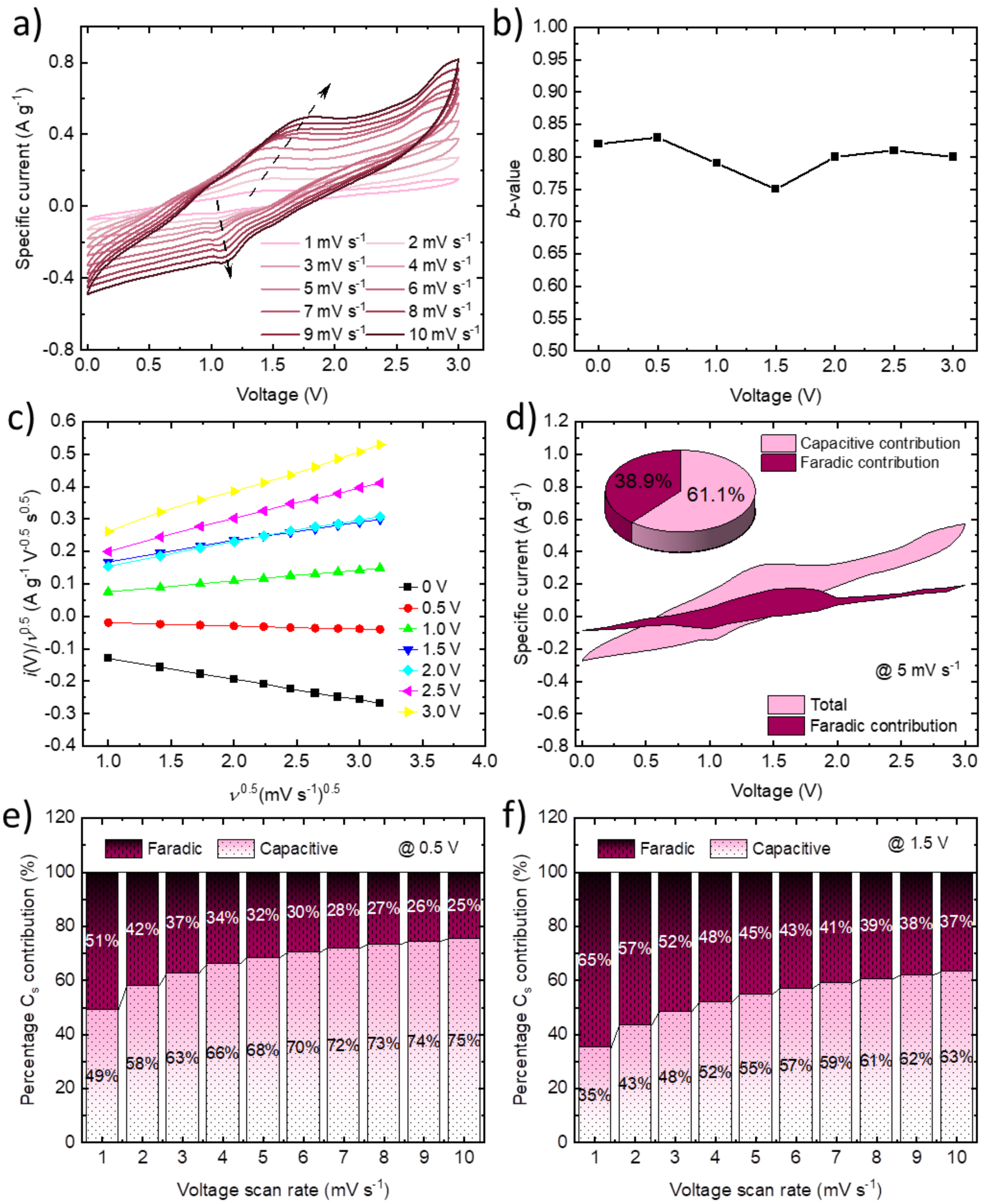

Figure S17. Charge storage mechanism of AC//p-HHB-Cu-LiPF$_6$ HSC. a) CV curves measured at voltage scan rates ranging from 1 to 10 mV s^{-1} and b) corresponding b-values as a function of the voltage. c) The linear relation of i(V)/v$^{0.5}$ vs. v$^{0.5}$ at various voltages. d) Capacitive and (Faradaic) diffusion-controlled currents determining the overall charge storage at 5 mV s^{-1}. Percentage Cs contribution of capacitive and Faradaic (diffusion-controlled) processes at e) 0.5 V and f) 1.5 V.

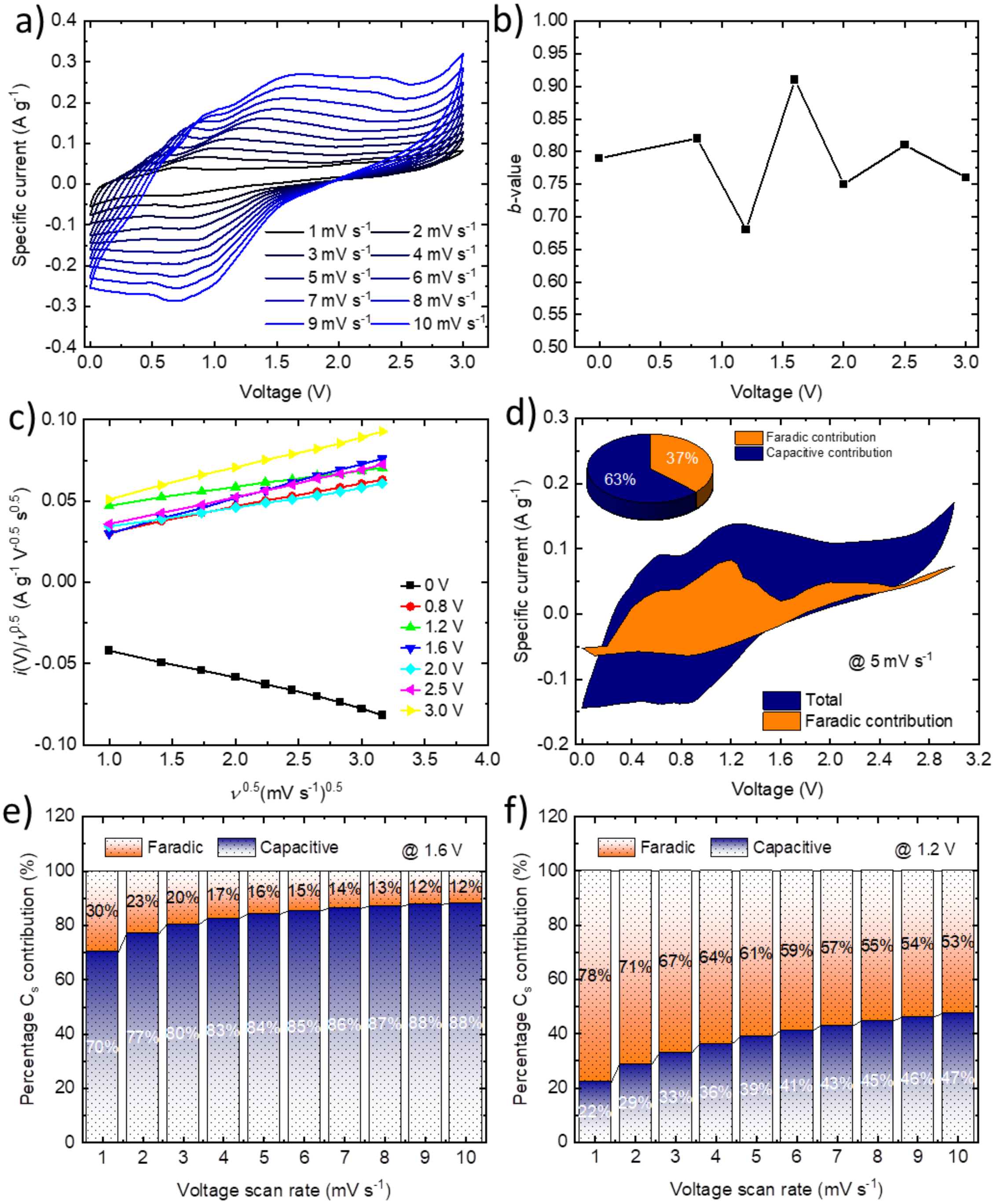

Figure S18. Charge storage mechanism of AC//np-HHB-Cu-LiPF$_6$ HSC. a) CV curves measured at voltage scan rates ranging from 1 to 10 mV s^{-1} and b) corresponding b-values as a function of the voltage. c) The linear relation of i(V)/v$^{0.5}$ vs. v$^{0.5}$ at various voltages. d) Capacitive and (Faradaic) diffusion-controlled currents determining the overall charge storage at 5 mV s^{-1}. Percentage Cs contribution of capacitive and Faradaic (diffusion-controlled) processes at e) 1.6 V and f) 1.2 V.

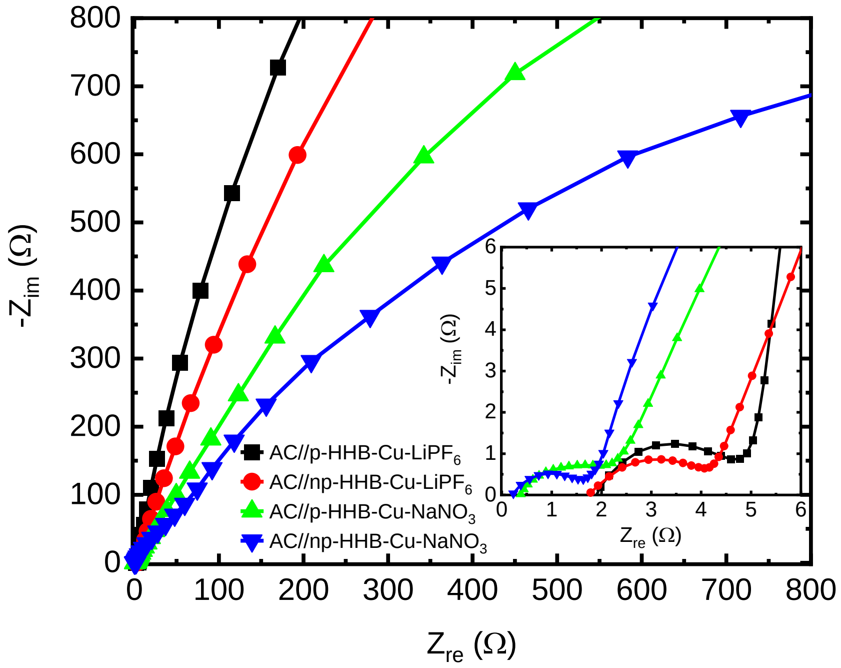

Figure S19. Nyquist plots measured for HHB-Cu-based HSCs; the inset shows the magnified view of the high-frequency region.

References

[1] Z. Wang, G. Wang, H. Qi, M. Wang, M. Wang, S. W. Park, H. Wang, M. Yu, U. Kaiser, A. Fery, S. Zhou, R. Dong, X. Feng, *Chem. Sci.* **2020**, *11*, 7665.
